# A draft genome, resequencing, and metabolomes reveal the genetic background and molecular basis of the nutritional and medicinal properties of loquat (*Eriobotrya japonica* (Thunb.) Lindl)

**DOI:** 10.1038/s41438-021-00657-1

**Published:** 2021-11-01

**Authors:** Yunsheng Wang

**Affiliations:** School of Life and Health Science, Kaili University, Kaili City, Guizhou Province 556011 China

**Keywords:** Genomics, Secondary metabolism

## Abstract

Loquat (*Eriobotrya japonica*) is a popular fruit and medicinal plant. Here, a high-quality draft genome of the *E. japonica* ‘Big Five-pointed Star’ cultivar that covers ~98% (733.32 Mb) of the estimated genome size (749.25 Mb) and contains a total of 45,492 protein-coding genes is reported. Comparative genomic analysis suggests that the loquat genome has evolved a unique genetic mechanism of chromosome repair. Resequencing data from 52 loquat cultivars, including 16 white-fleshed and 36 yellow-fleshed variants, were analyzed, and the flower, leaf, and root metabolomes of ‘Big Five-pointed Star’ were determined using a UPLC-ESI-MS/M system. A genome-wide association study identified several candidate genes associated with flesh color in *E. japonica*, linking these phenotypes to sugar metabolism. A total of 577 metabolites, including 98 phenolic acids, 95 flavonoids, and 28 terpenoids, were found, and 191 metabolites, including 46 phenolic acids, 33 flavonoids, and 7 terpenoids, showed no differences in concentration among the leaves, roots, and flowers. Candidate genes related to the biosynthesis of various medicinal ingredients, such as phenolics, flavonoids, terpenoids, and polysaccharides, were identified. Some of these genes were confirmed to be members of expanding gene families, suggesting that the high concentrations of beneficial metabolites in loquat may be associated with the number of biosynthetic genes in this plant. In summary, this study provides fundamental molecular insights into the nutritional and medical properties of *E. japonica*.

## Introduction

*Eriobotrya japonica* (Maloideae: Rosaceae), commonly known as loquat, is a type of evergreen fruit crop with a delicious taste and high nutrient contents^1^. According to documentary records and archaeological relics, *E. japonica* was first domesticated during the Han dynasty in China, 2000 years ago^2^. Today, it has been planted in more than 30 countries, including Japan, the United States, France, Italy, Egypt, and Spain. Its annual yield exceeds 1.2 million tons worldwide^3^. *E. japonica* cultivars can be divided into two groups based on their pulp and pericarp color: white- or yellow-fleshed. White-fleshed cultivars have higher sucrose contents than their yellow-fleshed counterparts, making them taste better. Yellow-fleshed fruit cultivars, which are the dominant cultivars, have higher nutritional value than their white-fleshed counterparts due to increased carotene contents^4^. *E. japonica* is also an important medicinal plant; its roots, leaves, and flowers have long been used in traditional Chinese medicine for the treatment of inflammation, diabetes, cancer, bacterial infection, aging, pain, and allergy^5–7^.

Whole-genome sequencing has been performed on other important fruit-producing crops and ornamental plants in the Rosaceae family, including *Malus domestica*^8^, *Prunus persica*^9^, *Pyrus bretschneideri*^10^, *Pyrus betuleafolia*^11^, *Fragaria vesca*^12^, *Prunus mume*^13^, *Prunus avium*^14^, *Prunus yedoensis*^15^, *Rubus occidentalis*^16^, *Fragaria ananassa*^17^, and *Rosa multiflora*^18^. The corresponding genomic data have provided global genetic information associated with their growth and development, ecological adaptive, and horticultural traits, which has been invaluable for breeding new varieties and tracking the complex evolution of these species. An increasing number of medicinal plants have appeared on the list of genome-sequenced species, which has significantly enhanced our understanding of the genetic background and molecular basis associated with the biosynthesis of medicinal components by these plants^19–22^.

As a popular fruit and medicinal plant, *E. japonica* has received particular attention from scientists and is the subject of horticultural, biological, and pharmaceutical research. However, there remains a gap in our knowledge regarding the genetic background of *E. japonica*, particularly with respect to the molecular basis of medicinal compound biosynthesis. Clarifying this issue would undoubtedly represent important progress in our understanding of the molecular pharmacognosy of *E. japonica*.

Here, a draft genome of the ‘Big Five-pointed Star’ yellow-fleshed *E. japonica* cultivar was assembled and annotated. Resequencing data from 52 *E. japonica* cultivars, including 16 white-fleshed and 36 yellow-fleshed cultivars, were analyzed, and the metabolite profiles of leaf, flower, and root tissues from the ‘Big Five-pointed Star’ cultivar were determined. The major aims of this study were to construct an additional high-quality reference genome for further research and utilization and to evaluate and provide insights into the nutritional and medicinal properties of *E. japonica*.

## Results

### Sequencing and assembly of a high-quality loquat draft genome

An individual ‘Big Five-pointed Star’ plant was selected for sequencing. Approximately 688.18 million clean short reads and a total of 51.54 Gb of data were generated using the HiSeq 4000 sequencing platform (Illumina, San Diego, CA, USA) (Table S1). These data were combined with a k-mer analysis to estimate a ‘Big Five-point Star’ genome size of 749.25 Mb (Table S2; Fig. S1), which was almost identical to that (749 Mb) determined using flow cytometry^23^, and the heterozygosity and GC content of the genome were found to be 0.31% and 38.58%, respectively (Table S2). Whole-genome sequencing was then performed using PacBio long-read sequencing technology (Pacific Biosciences, Menlo Park, CA, USA), and more than six million clean subreads with an average length of 6121 bp (N50 = 11,469 bp) were obtained (Table S3, Fig. S2). With these clean subreads, an initial draft genome composed of 3677 contigs with 733.32 Mb of nonredundant sequences was assembled (Table S4), covering ~97.87% of the estimated genome size. Three measures were adopted to evaluate the completeness of the initial draft genome assembly. First, the screening of 458 core eukaryotic genes and 248 conserved sequence datasets in the Core Eukaryotic Genes Mapping Approach (CEGMA) database^24^ identified 447 (97.06%) and 238 (95.97%) matches, respectively (Table S5). Second, using the Benchmarking Universal Single-copy Orthologs (BUSCO) database^25^, which contains 2326 plant-specific orthologous genes, a total of 2170 (93.29%) genes were identified, among which 1450 were single, 689 were duplicated complete, and 31 were fragmented. The number of missing BUSCO genes was only 156 (6.71%) (Table S5). Last, by mapping the short-read data onto the draft genome, it was found that 93.81% of the draft genome could be aligned (Table S5). The above results suggested that the initial draft genome had good assembly completeness. Approximately 96 Gb of data from ~321.2 million reads generated on the Illumina HiSeq 4000 sequencing platform were used to locate the contigs on chromosomes with Hi-C technology (Table S6). Among these reads, ~159.7 million read pairs were uniquely mapped to the initial draft genome, and more than 74.6 million read pairs were shown to represent valid interactions (Table S6). These read pairs were used to scaffold the contigs onto 17 chromosomes (Fig. S3), and the number of contigs was finally corrected to 3938, among which 3725 contigs (727.40 Mb, covering 99.19% of the draft genome sequence) were anchored to chromosomes. The order and direction on the chromosomes of 2181 contigs (644.88 Mb) could be determined (Table S7). These results indicate that the final assembled draft genome had good integrity and can be employed as reference whole-genome resequencing data and for other purposes.

### Genome element annotation

**Fig. 1:**
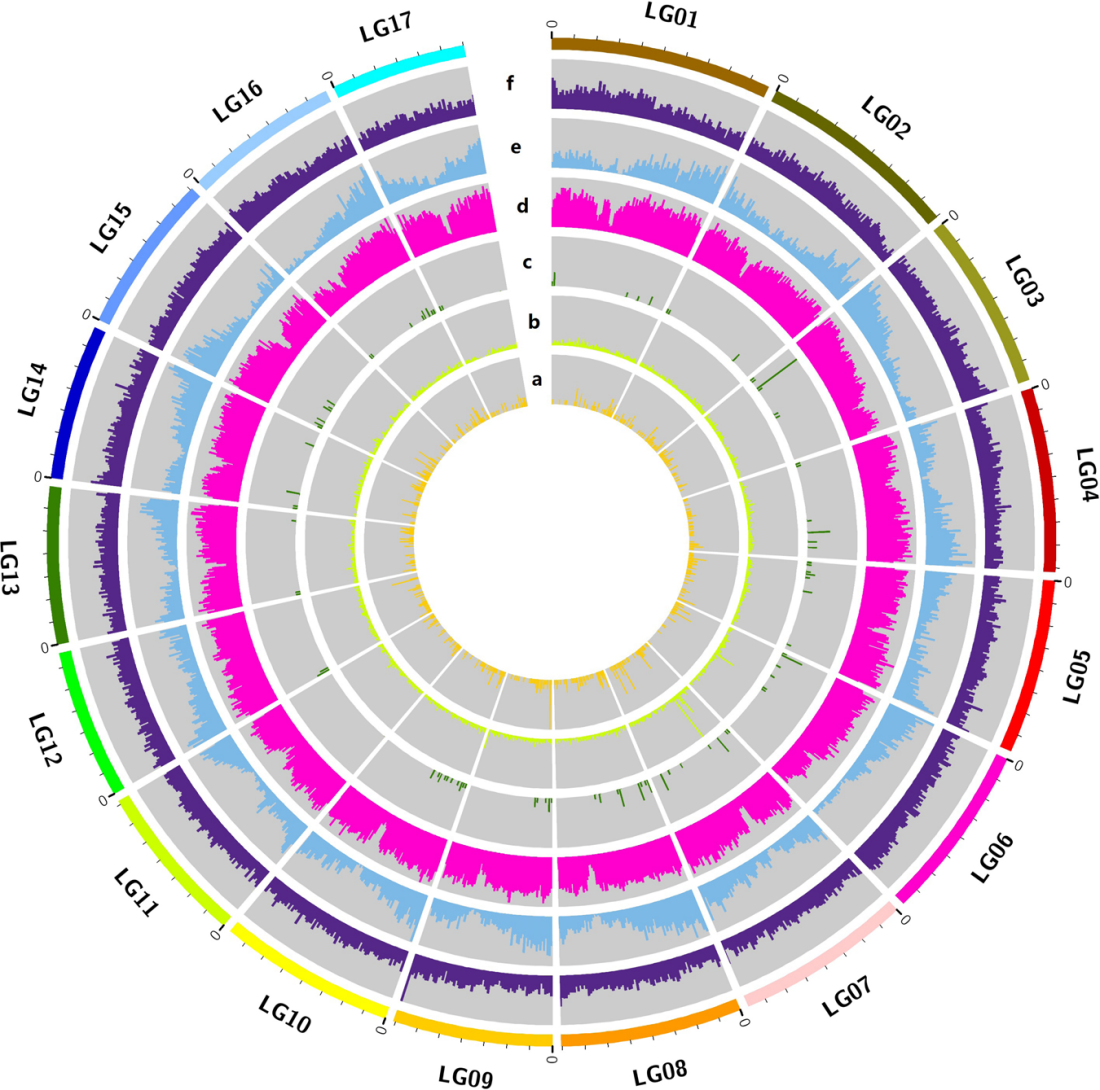
Characterization of the *E. japonica* genome. The number and length (Mb) of the pseudochromosomes are indicated outside the ring. Note: **a**, tRNA; **b**, rRNA; **c**, miRNA; **d**, repetitive sequences; **e**, genes, and **f**, GC content

Approximately 516.11 Mb of repetitive sequences were identified in the *E. japonica* draft genome, accounting for 70.38% of all sequences (Table S8). These repetitive sequences, in addition to 1.22 Mb (0.23%) of potential host gene sequences, mainly comprised transposable elements, including 423.60 Mb of RNA retrotransposons (Class I) and 113.40 Mb of DNA transposons (Class II) (Table S8). Copia and Gypsy long terminal repeats were shown to be the major types of RNA retrotransposons, constituting 158.90 Mb (30.79%), and 204.74 Mb (39.67%), respectively, of the total repetitive sequences (Table S8). The terminal inverted repeat (TIR) type accounted for the majority of the repetitive DNA transposon sequences, constituting 88.28 Mb (17.10%) of the total repetitive sequences (Table S8). By integrating *de novo* prediction, homologous species prediction, and transcriptome prediction to determine protein-coding genes in nonrepetitive regions of the draft genome, a total of 45,492 protein-coding genes, with an average length of 3420 bp and an average exon length of 1532 bp, were identified (Table S9, 10). Among these genes, 45,090 (99.12%) could be annotated (Table S11; Supplementary data file 1). In addition, 10,426 rRNA genes belonging to four different families, 165 miRNA genes belonging to 25 families, 691 tRNA genes belonging to 24 families, 197 snRNAs, 1023 snoRNAs, and 8314 pseudogenes were also identified in the final assembled draft genome (Table S12; Supplementary data file 2). The distribution pattern of protein-coding genes and RNA genes on the chromosomes was very uneven (Fig. 1).

The *E. japonica* genome contained a relatively high proportion of repetitive sequences and large numbers of protein-coding and rRNA genes. Nevertheless, the gene density was lower than in the other sequenced diploid Rosaceae species (Table 1). A high degree of correlation was observed among genome size, repetitive sequence length (*r* = 0.98, *p* < 0.05), and putative protein-coding gene numbers in all nine species (*r* = 0.97, *p* < 0.05). These results provide statistical evidence that repetitive sequences are the major determinants of genome size in Rosaceae species; the expansion of genome size may be accompanied by an increased gene number in the Rosaceae lineage.

**Table 1:** Summary of genome components of common loquat and nine other diploid sequenced Rosaceae species

Species	GS (Mb)	DGS (Mb)	DGS/GS (%)	RGS (Mb)	RGS/DGS (%)	TEs (Mb)	TEs/DGS (%)	Non RGS (Mb)	PG No.	GD No./10Kb	miRNA/tRNA/rRNA/snRNA/snoRNA	Reference
*Eriobotrya japonica*^a^	749.3	733.0	97.8	516.1	70.4	514.9	70.2	216.9	45,450	0.62	165/691/10,426/197/1023	–
*Malus × domestica*	742.3	603.9	81.3	352.6	58.4	314.5	52.1	241.6	57,386^b^	0.78	178/982/3508/346/287	8
*Prunus persica*	265.0	227.4	85.8	84.4	37.1	67.3	29.6	143.0	27,852	1.05	189/474/949/769/-	9
*Pyrus betuleafolia*	527.0	512.0	97.2	271.9	53.1	253.4	49.5	240.1	42,812	0.81	279/1148/697/395/	10
*Fragaria vesca*	240.0	209.8	87.4	48.1	22.9	46.7	19.2	161.7	34,809	1.45	76/569/177/168	12
*Prunus mume*	280.0	237.0	84.6	106.8	45.1	104.6	44.1	130.2	31,390	1.12	209/508/125/287	13
*Prunus avium*	352.9	272.4	77.8	119.4	43.8	43.1	19.0	153.0	43,349	1.23	−/365/71/-/-	14
*Prunus yedoensis*	257	323.8	126.0	150.8	46.6	127.1	39.3	173.0	41,294	1.61	–	1
*Rubus occidentalis*	293	242.9	82.9	136.1	56.0	130.7	53.8	106.8	32,300	1.10	–	16
*Rosa chinensis*	532.7	512.0	96.1	279.6	54.6	249.3	48.7	232.4	36,377	0.68	99/751/186/170/384	18
*Eriobotrya japonica*^c^	710.8	760.1	106.9	449.7	59.2	442.8	58.3	310.4	45,743	0.64	121/656/6,211/-/-	29

*GZ* genome size, *DGZ* draft genome size, *RGS* repetitive genome size, *TEs* transposable elements, *PG* protein-coding genes, *GD* gene density, – indicates no applicable data

^a^Big five-pointed star

GZ: ^b^Wu et al.^10^ reanalyzed the apple genome sequence and corrected the protein-coding gene number to 45,293

^c^Seven star

### Unique molecular mechanisms underlying genome recombination and repair in *E. japonica*

To understand the evolutionary pattern of the *E. japonica* genome, a comparative genomics analysis was performed using eight sequenced diploid Rosaceae species, including apple (*M. domestica*), pear (*P. betuleafolia*), peach (*P. persica*), sweet cherry (*P. avium*), Chinese plum (*P. mume*), black raspberry (*R. occidentalis*), woodland strawberry (*F. vesca*), and rose (*R. chinensis*), and the model plant *Arabidopsis thaliana*. The protein-coding genes of all ten species were clustered into gene families according to sequence similarity, and a total of 34 895 families were classified (Supplementary data file 3). Among these families, 544 families containing 1632 genes were shown to be unique to *E. japonica* (Supplementary data file 4). Gene Ontology (GO) enrichment analysis showed that these genes were primarily involved in biological process categories such as ‘DNA integration’, ‘RNA-intended biological process’, ‘DNA recombination’ and ‘DNA metabolism’, and in molecular functions, such as ‘RNA-directed DNA polymerase activity’, ‘RNA-DNA hybrid ribonuclease activity’ and ‘RNA binding’. In addition, Kyoto Encyclopedia of Genes and Genomes (KEGG) enrichment annotation showed that these *E. japonica* genes were primarily involved in metabolic pathways including ‘homologous recombination’, ‘base excision repair’, ‘nucleotide excision repair’, ‘DNA replication’, and ‘mismatch repair’ (Fig. S4; Supplementary data file 4). These results imply that *E. japonica* may have evolved unique genetic and molecular mechanisms for genome recombination and repair.

### Loquat originated earlier than apple and pear according to phylogenomics

**Fig. 2:**
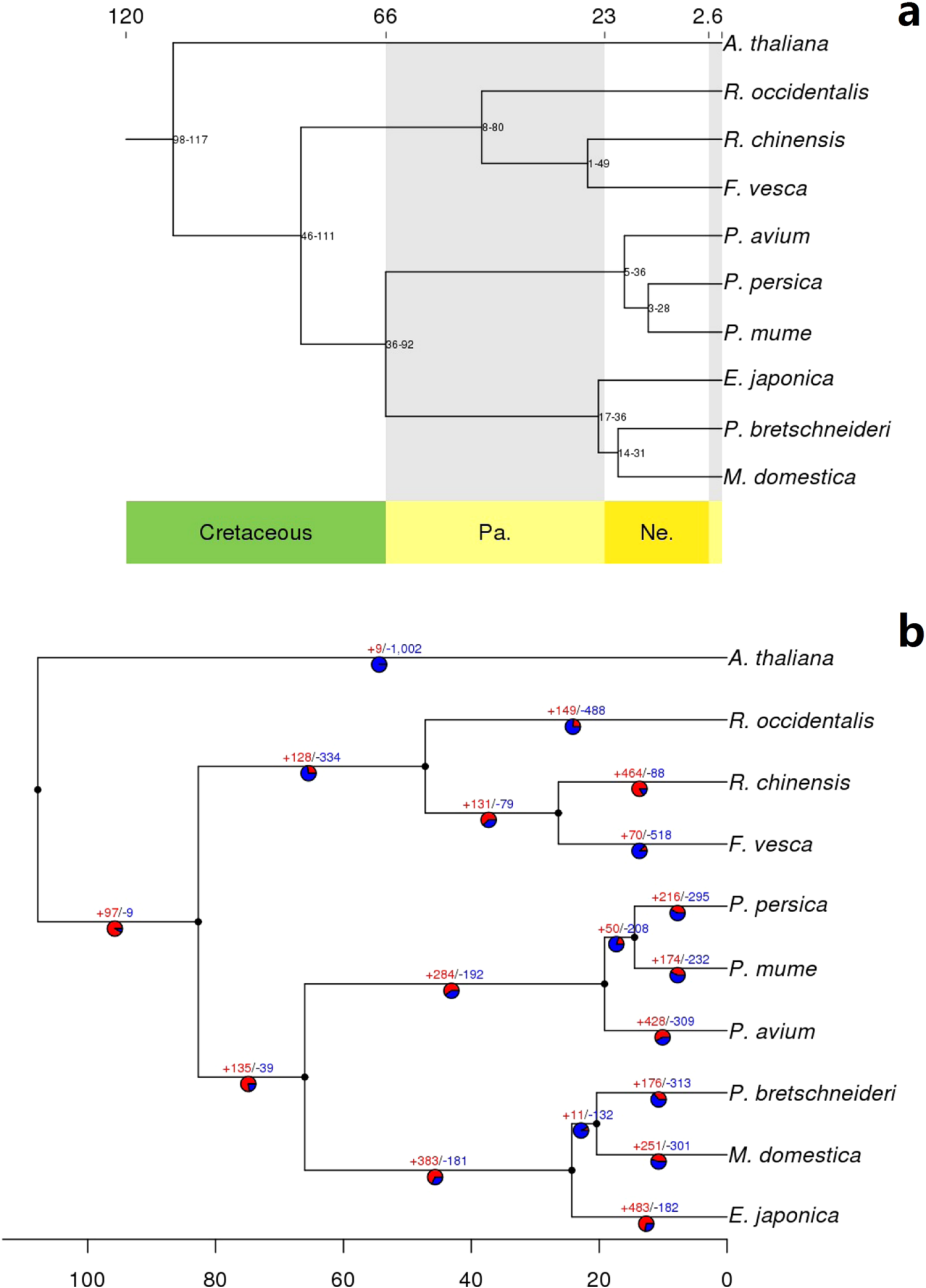
Phylogenetic tree of the nine sequenced diploid Rosaceae species and *Arabidopsis* contructed using 594 single-copy orthologs. **a**, Evolutionary relationship and divergence time; **b**, Gene family expansion and contraction on each evolutionary branch. Note: Red numbers preceded by a positive sign indicate the number of expanded gene families, and the numbers in blue preceded by a negative sign represent the number of contracted gene families

Apple, pear, and loquat all belong to the Amygdaloideae subfamily. However, the phylogenetic relationships between these three species have not been determined^26,27^. Here, a phylogenetic tree (Fig. 2a) containing nine Rosaceae species with *A. thalian*a as the outgroup was constructed using the protein sequences of 594 single-copy gene families (Supplementary data file 5). The analysis placed loquat, pear, and apple at the distal end of the outgroup, suggesting that the speciation time of the Amygdaloideae lineages was relatively late. The topological position of the nine Rosaceae species in the phylogenetic tree was consistent with that indicated by a previous study by Xiang et al.^26^. Molecular clock analysis indicated that the loquat lineage originated ~23 million years ago (MYA), with a 95% confidence interval of ~17–36 MYA, which is close to the beginning of the Neogene Period of the Cenozoic Era. All species of the Rosaceae family separated from the common ancestor ~82 MYA (95% confidence interval: ~46–111 MYA,) in the Late Cretaceous period, consistent with a previous dating analysis by Forest & Chase^28^. These results describe the time and order of species differentiation and differ from the data described by Jiang et al.^29^, which put pear before loquat and indicated that the Amygdaloideae subfamily originated ~8.6 MYA. The most likely reason for these differences was the different data used to construct the phylogenetic trees. Here, 594 single-copy gene sequences were used, while only 51 single-copy genes were used in the study by Jiang et al.^29^.

### Genes enriched for genetic repair functions have undergone positive selection

Further investigations of the single-copy gene families were then completed to establish if any family was under positive selection. Among the 594 single-copy gene families used to construct the phylogenetic tree, 90 (15.15%) families from *E. japonica* were found to have undergone strong positive selection. These genes were mainly enriched for functions including ‘metabolic process’ and ‘cellular process’ in the ‘biological process’ category, ‘cell part’, ‘cell’ and ‘organelle’ in the ‘cellular component’ category, and ‘catalytic activity’ and ‘binding activity’ in the ‘molecular function’ category (Fig. S5a; Supplementary data file 6). These genes were also found to be enriched in metabolic pathways such as ‘nucleotide excision repair’ (Fig. S5b; Supplementary data file 6). These results provide additional genetic evidence that *E. japonica* has evolved a unique set of DNA repair and recombination mechanisms.

### Expanded gene families linked to both medicinal compound biosynthesis and fruit flavor

A total of 483 gene families with significant expansion were detected in the *E. japonica* genome (Fig. 2b). These included 4472 genes in total, which were most enriched for GO terms, in the ‘biological process’ (‘metabolic process’ and ‘cellular process’), ‘cellular component’ (‘cell part’, ‘cell’, and ‘organelle’) and ‘molecular function’ (‘catalytic activity’ and ‘binding activity’) categories. KEGG analysis indicated that these genes were enriched for metabolic pathways involving ‘monoterpenoid biosynthesis’ and ‘starch and sucrose metabolism’ (Fig. S6; Supplementary data file 7). These results suggest a change in the genetic mechanism related to the metabolism of terpenoids and soluble polysaccharides in *E. japonica*.

### Genome-wide association study (GWAS) of flesh color

**Fig. 3:**
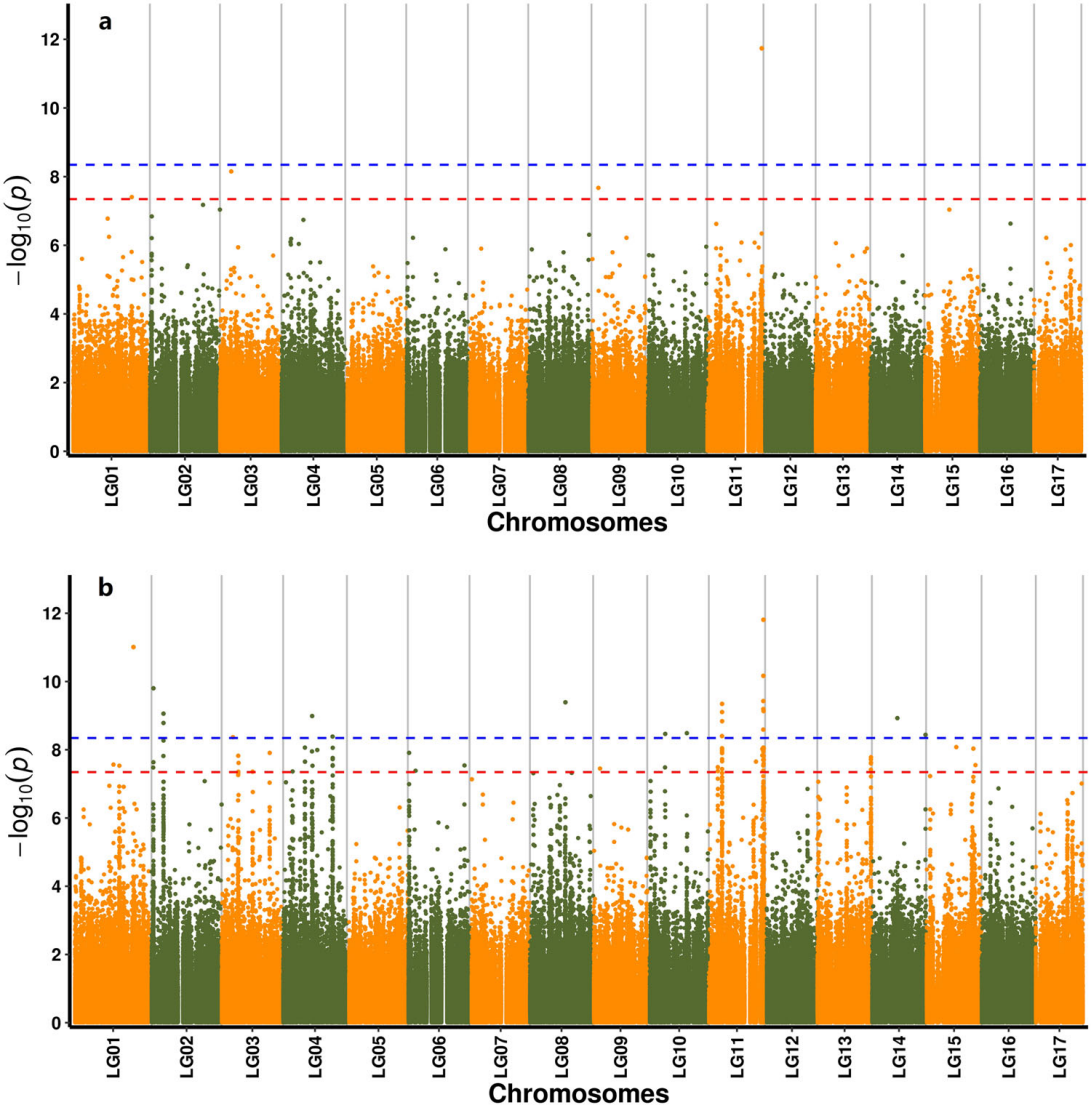
Manhattan plot of GWAS. **a**, Results by EMMAX; **b**, Results by FaST-LMM. Note: The dashed lines indicate significant correlations at *P* = 0.05 and 0.01, respectively

Flesh color is one of the most important agricultural characteristics of *E. japonica* and other fruit crops. A GWAS for flesh color traits was performed using the EMMAX program with an efficient mixed-model association based on a linear mixed model^30^ and the linear mixed model program FaST-LMM^31^. EMMAX analysis identified four single nucleotide polymorphism (SNP) loci that were significantly associated with flesh color (*p* < 0.005) on chromosomes 1, 3, 9, and 11, including 70 gene loci located within 100 kb of these SNPs (Table S13; Fig. 3a; Fig. S7a; Supplementary data file 8). Fastlmm analysis identified 22 SNPs significantly associated with flesh color (*p* < 0.001) on chromosomes 1 (*n* = 1), 2 (*n* = 3), 3 (*n* = 1), 4 (*n* = 2), 8 (*n* = 1), 10 (*n* = 2), 11 (*n* = 10), and 14 (*n* = 2), with 232 gene loci located within 100 kb of these SNPs (Table S13; Fig. 3b; Fig. S7b; Supplementary data file 3). These results suggest that more genomic regions of chromosome 11 have undergone selection for flesh color determination in *E. japonica* relative to other chromosomes. SNP loci on chromosomes 1, 3, and 11, but not on chromosome 9, were detected by both EMMAX and Fastlmm (*p* < 0.001) (*p* < 0.001) (Supplementary data file 8). Among the identified genes, *EVM0006083.1* (encoding GDP-mannose 4,6 dehydratase 1), *EVM0031803.1* (encoding glucuronosyltransferase PGSIP6), *EVM0034038.1* (encoding glucose-6-phosphate 1-epimerase), *EVM0034751.1* (encoding probable beta-1,3-galactosyltransferase 11), and *EVM0040993.1* (encoding sucrose transport protein SUC3 isoform X1) were related to sugar metabolism. Notably, the *EVM0031803.1* gene was located within 100 kb of multiple significant SNPs (Supplementary data file 8).

### Metabolite profiles of the flowers, leaves, and roots of the *E. japonica* tree and their KEGG pathway annotation

**Fig. 4:**
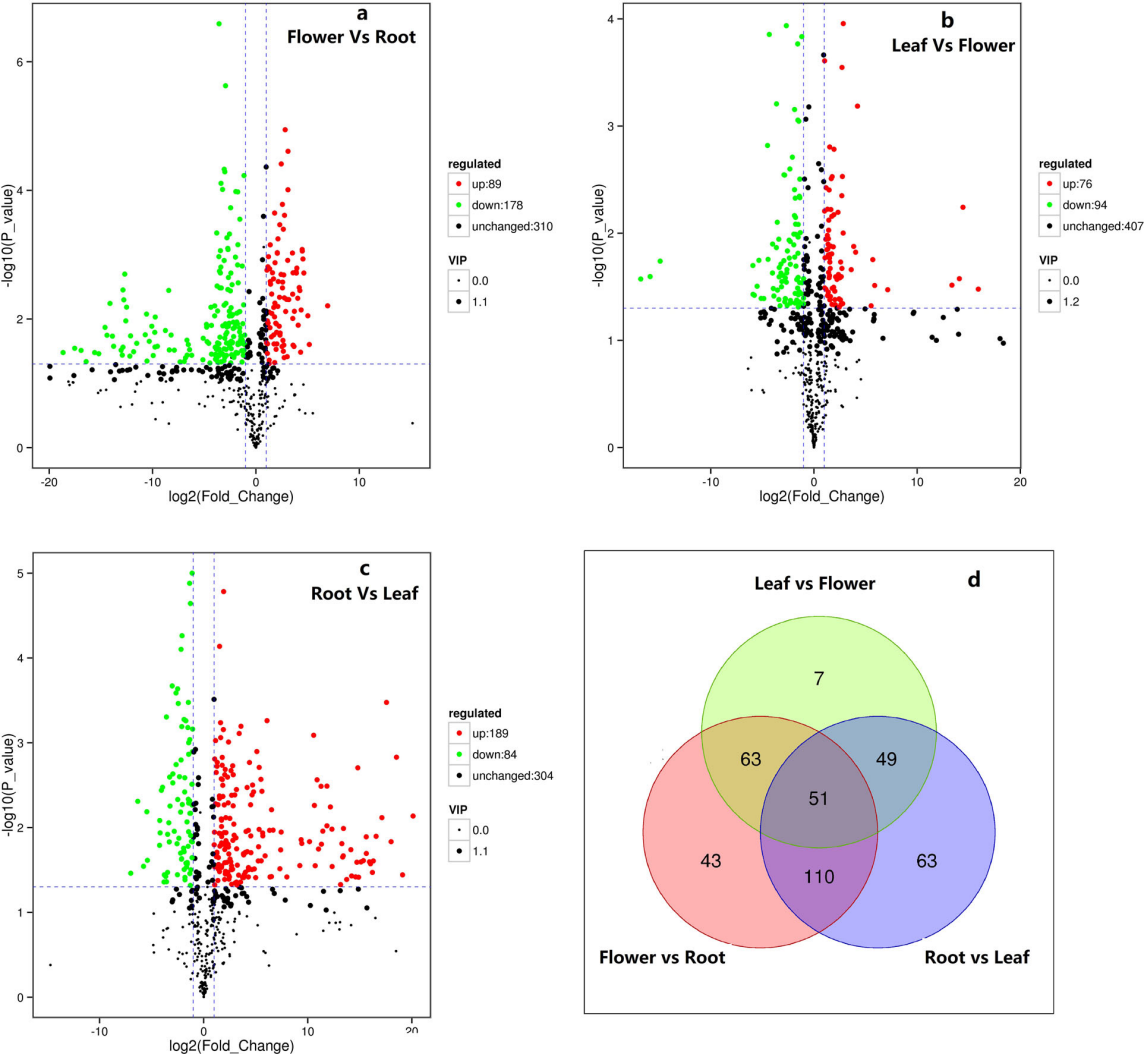
Heatmap of metabolites demonstrating the differences in their overall expression. **a**, Difference between the flower and root; **b**, Difference between root and leaf; **c**, Difference between leaf and flower; **d**, Venn statistics for all three pairs

Principal component analysis and Pearson’s correlation analysis of the metabolite data obtained from nine samples revealed a high degree of correlation among the metabolites obtained from the same organ (Figs. S8, S9), confirming that the mass spectrometry data from these samples were reliable and that the metabolite profiles of the same organ from different samples were more similar than those from different organs from the same *E. japonica* plant. A total of 577 metabolites were detected in total, including 193 phenols, 33 alkaloids, 28 terpenoids, and one steroid. More metabolites (573) were found in the flowers than in the leaves (565) or roots (509) (Table 2; Supplementary data file 9, 10). This is in reasonable agreement with the 536 metabolites, including 60 organic acids, identified in a previous study of *E. japonica* fruits^32^. More metabolites and fewer organic acids were found in *E. japonica* leaves and flowers, respectively. The flowers contained 89, 178, and 310 metabolites that were upregulated, downregulated, and unchanged, respectively, compared to those in the root metabolome (Fig. 4a; Supplementary data file 11). These values were 76, 94, and 407 when the flower metabolome was compared with that of the leaf (Fig. 4b; Supplementary data file 12) and 189, 84, and 304 when comparing the leaf and root metabolomes, respectively (Fig. 4c; Supplementary data file 13). There were significant differences in the quantities of 51 metabolites among the leaves, flowers, and roots; however, there were no significant differences in 192 metabolites between these organs (Fig. 4d; Supplementary data file 14). These results demonstrated that the *E. japonica* flowers, roots, and leaves were all rich in metabolites, many of which did not show significant differences in accumulation among these three organs, which could explain why *E. japonica* roots, leaves, and flowers all have medicinal value. A total of 271 of these metabolites could be annotated using the KEGG database (Supplementary data file 9, 15). However, only 110 metabolites, including 15 phenolic acids, 6 flavonoids, and 12 alkaloids, could be assigned to specific pathways, such as ‘metabolic pathways’ (ko01100), ‘biosynthesis of secondary metabolites’ (ko01110), ‘phenylpropanoid biosynthesis’ (ko00940), ‘flavonoid biosynthesis’ (ko00941), ‘stilbenoid, diarylheptanoid and gingerol biosynthesis’ (ko00945), and ‘isoflavonoid biosynthesis’ (ko00950) (Supplementary data file 10).

**Table 2:** Statistics on metabolites in loquat flowers, leaves, and roots

Class I	Class II	All	Flower	Leaf	Root
Amino acids and derivatives	Amino acids and derivatives	51	51	49	46
Nucleotides and derivatives	Nucleotides and derivatives	48	48	48	45
Organic acids	Organic acids	36	36	36	35
Lipids	Free fatty acids	28	28	28	27
	Glycerol ester	27	27	27	25
	Lysophosphatidylcholine	20	20	20	20
	Lysophosphatidylethanolamine	18	18	17	12
	Phosphatidylcholine	1	1	1	1
Others	Others	34	34	33	33
	Saccharides and alcohols	31	31	31	31
	Vitamin	8	8	8	8
Phenolic acids	Phenolic acids	98	95	96	85
	Anthocyanins	2	2	2	2
	Biflavones	1	1	1	1
	Chalcones	2	2	2	2
	Dihydroflavone	5	5	5	3
	Dihydroflavonol	3	3	3	2
	Flavanols	10	10	10	10
	Flavonoid	30	30	29	22
	Flavonoid carbonoside	3	3	3	3
	Flavonols	38	38	36	28
	Isoflavones	2	2	2	1
	Coumarins	4	4	4	4
	Lignans	10	10	10	10
	Tannins	3	3	3	3
	Proanthocyanidins	12	12	12	12
Alkaloids	Alkaloids	19	19	18	16
	Phenolamine	7	7	5	5
	Plumerane	6	6	6	6
	Pyrrole alkaloids	1	1	1	1
Terpenoids	Sesquiterpenoids	2	2	2	1
	Triterpene	25	24	25	18
	Triterpene Saponin	1	1	1	1
Steroids	Steroidal saponins	1	1	1	0
In total		577	573	565	509

### Phenolic acids in *E. japonica* flowers, leaves, and roots and the genes associated with their biosynthesis

Previous studies have indicated that the principal components responsible for the medicinal value of *E. japonica* are their phenolics, terpenoids, and polysaccharides^33–35^. Phenolic compounds, the aromatic secondary metabolites that occur in plants, can be clustered into different families, including phenolic acids, flavonoids, lignans, coumarins, and tannins. They have considerable potential benefits for human health, including antiaging effects and reducing the risk of oxidative stress and inflammation related to chronic diseases^36,37^. Phenolic acids and flavonoids are reasonably abundant in the tissues of *E. japonica* trees^34^. This study detected a total of 98 phenolic acids, including 95 phenolic acids present in the *E. japonica* flower, 96 in the *E. japonica* leaf, and 85 in the *E. japonica* root (Table 2). Among these phenolic acids, nine, including methyl caffeate and benzoyl feruloyltartaric acid, were found to exhibit differences in accumulation among flower, leaf, and root tissues, while 46 phenolic acids, including p-coumaric acid, vanillin, 6-O-caffeoylarbutin, caffeic acid, dibutyl phthalate, isosalicylic acid, and O-glycoside, showed no significant difference in accumulation, among which caffeic acid, dibutyl phthalate, isosalicylic acid, and O-glycoside were present at particularly high levels in all three tissues (Supplementary data file 13). Chlorogenic acid, caffeic acid, and their analogs and derivatives are essential phenolic acids in plants and play a significant role in human health^33^. In this study, chlorogenic acid, caffeic acid, and 10 of their analogs and derivatives, including 5-O-caffeoylshikimic acid, 6-O-caffeoylarbutin, caffeic acid, chlorogenic acid, caffeic aldehyde, 3,4-dicaffeoylquinic acid, O-caffeoyl maltotriose, dicaffeoylquinic acid-glucoside, syringoyl caffeoylquinic acid, O-glucose, neochlorogenic acid (5-O-caffeoylquinic acid), and chlorogenic acid methyl ester, were found in the leaves, flowers, and roots of *E. japonica*, and one derivative, 4-O-(6’-O-glucosylcaffeoylglucosylferuloyl)-4-hydroxybenzyl alcohol, was found in the leaves and flowers but not in the roots. 6-O-caffeoylarbutin, caffeic acid, 5-O-caffeoylshikimic acid, chlorogenic acid, and neochlorogenic acid (5-O-caffeoylquinic acid) were present at particularly high levels in all three organs (Supplementary data file 9, 10). These results provide an explanation for the use of all three organs of *E. japonica* trees in folk medicine in China with an appreciable curative effect.

Phenylpropanoids play a central role in the biosynthesis of phenolic compounds^38^. Here, a total of 13 compounds, including p-coumaryl alcohol, L-phenylalanine, coniferaldehyde, caffeic acid, and coniferyl alcohol, and 286 predicted protein-coding genes were annotated within the metabolic pathway of ‘phenylpropanoid biosynthesis’ (Ko00940) (Supplementary data files 10, 16). Among these genes, 21 were found to cluster into six different gene families (OG0016412: *EVM0001669.1*; OG0000101: *EVM0005803.1*, *EVM0006273.1*, *EVM0019449.1*, *EVM0022718.1*, *EVM0042804.1*, *EVM0044744.1*; OG0000058: *EVM0006222.1*, *EVM0013101.1*, *EVM0016248.1*, *EVM0013956*, *EVM0018971*, *EVM0023734*, *EVM0026236*, *EVM0041423*, *EVM0045044*; OG0019775: *EVM0025581.1*, OG0028725: *EVM0026375.1*, OG0021299: *EVM0026790.1*, *EVM0044979.1*; OG0016856: *EVM0014121.1*, and *EVM0004668*) putatively encoding shikimate O-hydroxycinnamoyltransferase-like proteins [EC:2.3.1.133] (Figs. S10, S11; Supplementary data file 3, 16), These proteins play an important role in the biosynthesis of p-coumaroylshikimic acid and caffeoyl-CoA, which have been shown to exhibit a high degree of accumulation in *E. japonica* flower, leaf, and root tissues. An additional 23 genes, including *EVM0013751.1*, *EVM0021008.1*, *EVM0027237.1*, *EVM0025905.1*, *EVM0005818.1*, *EVM0006892.1*, *EVM0000333.1*, *EVM0043812.1*, *EVM0018908.1*, *EVM0033659.1*, *EVM0036617.1*, *EVM0010682.1*, *EVM0020673.1*, *EVM0045230.1*, *EVM0022960.1*, *EVM0033075.1*, *EVM0005987.1*, *EVM0022134.1*, *EVM0016420.1*, *EVM0025415.1*, *EVM0017461.1*, *EVM0020259.1* and *EVM0018483.1*, were identified in this study. However, only five (*EVM0025905.1*, *EVM0000333.1*, *EVM0018908.1*, *EVM0020673.1*, and *EVM0025415.1*) of these genes were clustered into an expanding gene family (OG0000253) that putatively encodes caffeic acid 3-O-methyltransferase [EC:2.1.1.68], which plays a key role in the biosynthesis of ferulic acid and sinapyl alcohol (Figure S10, S11; Supplementary data file 7). Both of these metabolites were detected in *E. japonica* flower, leaf, and root tissues (Supplementary data file 9, 10).

### Flavonoids produced by *E. japonica* flower, leaf, and root tissues and the genes associated with their biosynthesis

Flavonoids are an important group of phenolics. Quercetin-type flavonols (primarily quercetin glycosides) are the most abundant type of flavonoid molecules found in plants and are known for their wide range of biological activities, all of which promote human health^35^. Thus far, at least 16 flavonoids, including quercetin, isoquercitrin, rutin, hyperoside, and quercitrin, have been found in and isolated from *E. japonica* samples^39^. Here, a total of 81 flavonoids were identified in *E. japonica*, including 2 anthocyanins, 1 biflavone, 2 chalcones, 5 dihydroflavones, 3 dihydroflavonols, 10 flavanols, 15 flavonoids, 3 flavonoid carbonosides, 38 flavonols, and 2 isoflavones (Table 2; Supplementary data file 13), most of which are reported for the first time. Quercetin-3-O-(2-O-rhamnosyl)-galactoside, quercetin-3-O-neohesperidoside, quercetin-3-O-xylosyl(1 → 2)-galactoside, and quercetin-3-O-glucoside (isoquercitrin) showed significant differences in accumulation in the *E. japonica* leaves, flowers, and roots. However, the accumulation of dihydroquercetin (taxifolin), quercetin-3-O-(6”-trans-p-coumaroyl)-glucoside, quercetin-5-O-glucuronide, and quercetin-3-O-(2”-acetyl)-glucuronide was not significantly different among these three tissues (Supplementary data file 14).

KEGG annotation identified 71 predicted protein-coding genes likely to be involved in flavonoid biosynthesis in *E. japonica* (Ko00941) (Supplementary Data File 16). Quercetin, an important flavonoid that has been shown to modify eicosanoid biosynthesis (anti-prostanoid and anti-inflammatory responses)^40^, is abundant in *E. japonica* and exists primarily in the form of quercetin glycosides (Supplementary data file 9, 10). Three genes (*EVM0007289.1*, *EVM0040197.1*, and *EVM0018354.1*), putatively encoding a key enzyme in the quercetin biosynthesis pathway, flavonoid 3′-monooxygenase [1.14.13.21], were identified in the *E. japonica* genome (Figure S12, S13).

### Terpenoids in *E. japonica* leaves, roots, and flowers and their biosynthetic genes

In addition to phenolic compounds, terpenoids constitute another major class of active ingredients in *E. japonica*. In particular, ursolic acid and oleanolic acid are well-known terpenoids with strong demonstrated bioactivity with potential benefits for human health^34,41–43^. At least 14 triterpene acids have been isolated from the *E. japonica* leaf thus far, all of which show marked anti-inflammatory effects^44,45^. Here, 28 terpenoids (2 sesquiterpenoids, 25 triterpenes, and 1 triterpene saponin) were detected in the *E. japonica* leaves, flowers, and roots, including oleanonic acid, ursolic acid, and four derivatives thereof (2-hydroxyoleanolic acid, 2,3-dihydroxy 5(6),12(13)diene ursolic acid, 27,28-dicarboxyl ursolic acid, and ursolic acid-OCH_3_) (Fig. 4; Data files 3, 4). δ-Amyrenone, ursolic acid-OCH_3_, and medicagenic acid 3-O-GlcA-28-O-Rha(1,2)-Ara exhibited significant differences in accumulation between the leaves, flowers, and roots, whereas betulinic acid, ursonic acid, betulonic acid, oleanonic acid, maslinic acid, 24,30-dihydroxy-12(13)-enolupinol, and 2-hydroxyoleanolic acid showed no significant differences in accumulation. Ursonic acid, pomolic acid, asiatic acid, and caffeoyl hawthorn acid were present in particularly high concentrations (Table 2; Supplementary data file 14). This is the first study to identify certain terpenoids, such as ligupleurol geniposide and β-amyrenone, in *E. japonica*. These results support the application of *E. japonica* leaves, flowers, and roots in folk medicine in China, providing evidence of their curative effects.

KEGG annotation linked 92, 32, 56, and 37 putative protein-coding genes to biosynthetic pathways associated with the production of terpenoid backbones (Ko00900), monoterpenoids (Ko00902), diterpenoids (Ko00904), and sesquiterpenoid-triterpenoids (Ko00909), respectively (Figs. S14–S17; Supplementary data file 16). However, very few terpenoids could be annotated, so it is difficult to directly link the terpenoids with their corresponding biosynthetic genes using these data.

### Polysaccharides in *E. japonica* leaves, flowers, and roots and their biosynthetic genes

In addition to phenolic compounds, terpenoids, alkaloids, and polysaccharides have been shown to have important medicinal properties^46^. Here, 16 monosaccharides (D-(-)-threose, D-(-)-arabinose, D-arabitol, D-glucose, sedoheptulose, N-acetyl-D-glucosamine, 5-O-feruloyl-L-arabinose, melibiose, D-(+)-sucrose, D-(+)-trehalose, isomaltulose, turanose, solatriose, raffinose, D-(+)-melezitose, and panose) and two glycosides (galactinol and D(+)-melezitose O-rhamnoside) were found to show significant differences in accumulation in *E. japonica* flowers, leaves, and roots (Supplementary data files 9, 10). 5-O-Feruloyl-L-arabinose, solatriose, isomaltulose, D(+)-melezitose O-rhamnoside, D-(+)-melezitose, D-(+)-sucrose, and N-acetyl-D-glucosamine accumulated uniformly, with no significant differences between the leaves, flowers, and roots. D-glucose, D-(+)-trehalose, galactinol, isomaltulose, and turanose were shown to exhibit particularly high concentrations in *E. japonica* flowers, leaves, and roots (Supplementary data file 9, 10).

**Fig. 5:**
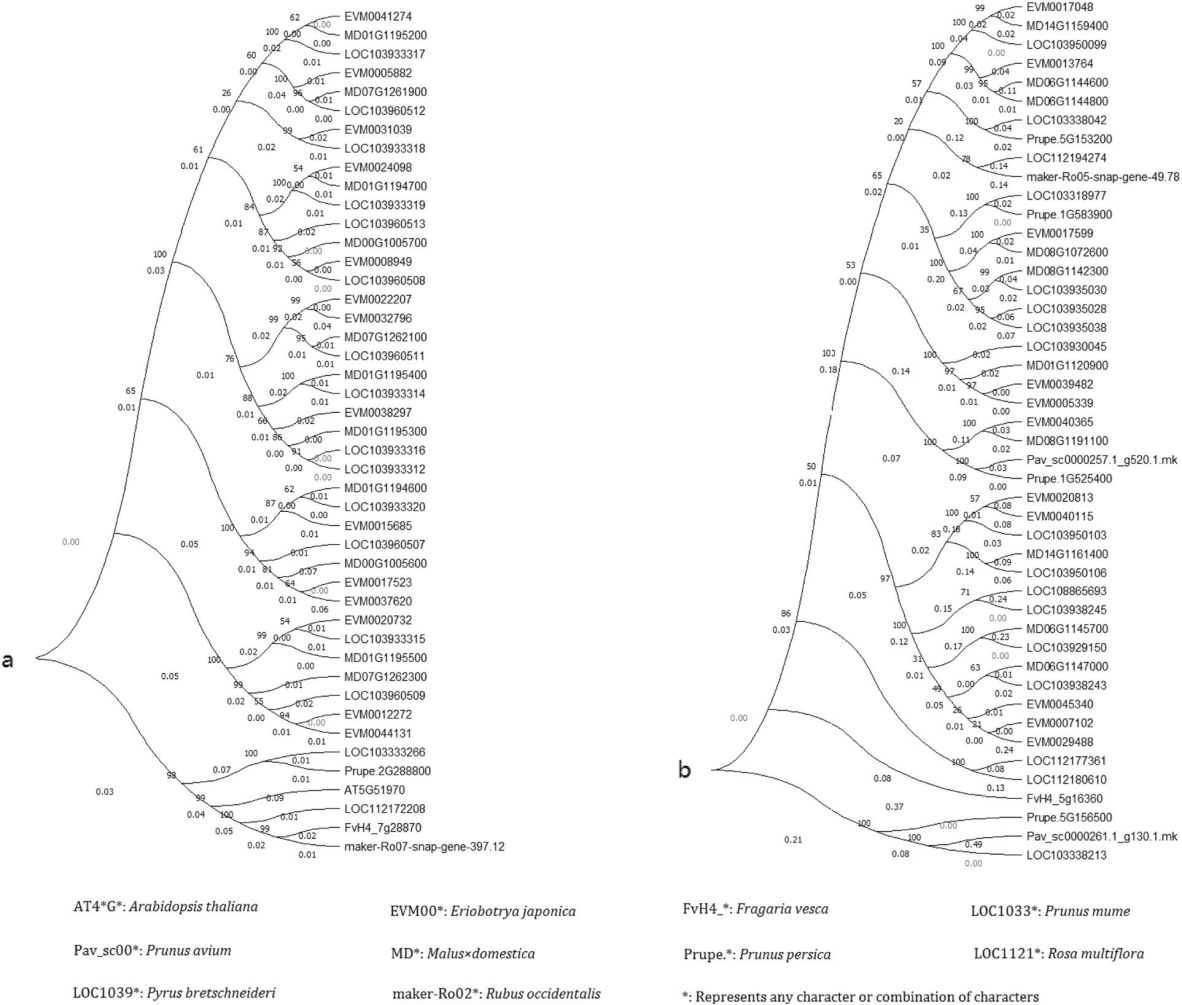
Phylogenetic analysis of gene families. **a**, OG0000179 gene family; **b**, OG0000175 gene family

Many *E. japonica* protein-coding genes were linked to various pathways associated with polysaccharide metabolism (e.g., 111 related to ‘fructose and mannose metabolism’ (Ko00051) and 92 related to ‘galactose metabolism’ (Ko00052)) (Supplementary data file 1). Among these genes, 11 (*EVM0005339*, *EVM0007102*, *EVM0013764*, *EVM0017048*, *EVM0017599*, *EVM0020813*, *EVM0029488*, *EVM0039482*, *EVM0040115*, *EVM0040365*, and *EVM0045340*) belonged to the OG0000175 gene family, which putatively encodes a beta-glucosidase 24-like protein [EC:3.2.1.21], and 14 (*EVM0005882*, *EVM0008949*, *EVM0012272*, *EVM0015685*, *EVM0017523*, *EVM0020732*, *EVM0022207*, *EVM0024098*, *EVM0031039*, *EVM0032796*, *EVM0037620*, *EVM0038297*, *EVM0041274*, and *EVM0044131*) belonged to the OG0000179 gene family, which putatively encodes a sorbitol dehydrogenase-like protein [EC:1.1.1.14]. EC:3.2.1.21 and EC:1.1.1.14 are important enzymes in the catalysis of D-glucose (Fig. S18) and D-fructose (Figure S19), respectively. Notably, both the OG0000179 and OG0000175 gene families have undergone expansion during their evolution (Supplementary data file 7). Phylogenetic trees of the OG0000179 and OG0000175 gene families revealed that some genes have undergone increases in in copy number only in the *E. japonica* genome. These *E. japonica*-specific replication pairs in the OG0000179 gene family included *EVM0017523* and *EVM0017523* and *EVM0012272* and *EVM0044131* (Fig. 5a). The OG0000175 gene family had similar replications in *EVM0039482* and *EVM0005339*; *EVM0020813* and *EVM0040115*; *EVM0007102*; and *EVM0029488* (Fig. 5b). In addition, D-glucose and many of its derivatives were found in the *E. japonica* flowers, leaves, and roots. At the same time, none of these tissues produced any fructose (Supplementary data file 9, 10), suggesting that fructose is only abundant in *E. japonica* fruit^32^. These results provide a meaningful link between the genetic background, such as the expansion of gene family members, and the accumulation of related metabolites.

## Discussion

In recent years, *E. japonica* has become an increasingly important fruit worldwide. However, due to the lack of information on its genome, studies on the genetics and molecular biology of *E. japonica* are limited. This situation is now changing, as a recent publication described a draft genome of the *E. japonica* ‘Seventh Star cultivar’^29^, and the present study describes a new high-quality draft genome of the *E. japonica* ‘Big Five-pointed Star’ cultivar. The ‘Big Five-pointed Star’ cultivar is known for its yellow/red flesh and has the largest cultivated area in China^47^. In contrast, ‘Seventh Star’ is a mutant cultivar with white flesh that was only recently bred into existence. Both the previous and current draft genomes show high assembly quality. In the former, 99.66% of the genome consisted of contig sequences, 89.27% of which were clustered into pseudochromosomes, as shown using Hi-C protocols. The corresponding statistics for this study are 99.19% and 87.94%, respectively. A comparative analysis showed that only three chromosomes in the draft genomes of the ‘Big Five-pointed Star’ and ‘Seventh Star’ cultivars present the same orientation and that 14 chromosomes could be considered complementary (Table S14). The predicted genome size and number of protein-coding genes in ‘Seventh Star’ are ∼710.83 Mb and 45 473, while those for ‘Big Five-pointed Star’ are ∼749.25 Mb and 45 492, respectively. This suggests active genome differentiation between different *E. japonica* varieties. The current *E. japonica* draft genome of ‘Big Five-pointed Star’, together with the predicted gene sequences, will be released to provide a greater variety *E. japonica* reference genomes for further molecular biology, genetic, and breeding studies.

Flesh color is an important horticultural and commodity trait that differs among *E. japonica* cultivars. This phenotype is controlled by the genotype but not by external environmental conditions, similar to many other horticultural and commodity traits. Flesh color is associated with markedly higher levels of colored carotenoids in the flesh tissue of red-fleshed cultivars than in the flesh tissue of white-fleshed cultivars^48^. The genetic basis of this difference has been investigated by different methods, including biochemical assays and functional genomics^49–51^, suggesting that different carotenoid types and contents lead to phenotypic differences in *E. japonica* flesh color. However, it is difficult to identify specific gene loci or alleles responsible for flesh color variations. GWAS offers a complementary and powerful tool for elucidating the relationship between genotype and phenotype^52,53^. Numerous studies have revealed substantial genotype–phenotype associations in crops, highlighting the value of GWAS in functional genomic studies^54^. This study identified a set of SNPs and alleles that are significantly associated with flesh color and contributed to the identification of potential candidate genes for phenotypes located near these SNPs in the genome. However, after careful testing, none of these genes were directly involved in the metabolism of carotenes. However, some of these genes were involved in sugar metabolism. These results provide several molecular clues linking sugar contents to the differing nutrient properties of red-fleshed and white-fleshed *E. japonica* cultivars.

Some important metabolites with potential medicinal value, including ursolic acid, ursolic acid methyl ester, acetyl ursolic acid, oleanolic acid, chlorogenic acid, neochlorogenic acid, and caffeic acid, have been detected in different organs of *E. japonica* using classical instrumental analyses^55,56^. However, traditional phytochemistry methods are time-consuming and labor-intensive and present a low throughput. There are limited data describing the metabolome of *E. japonica*, including its production of phenols, flavonoids, terpenes, and polysaccharides with potential health benefits. This lack of information is not conducive to further research on the medicinal properties of *E. japonica* and its utilization. In recent years, our collective understanding of traditional Chinese medicine has been considerably advanced by the use of various analytical technologies and genomics, proteomics, and metabolomics research^57,58^. Among these approaches, metabolomics has been particularly valuable for analyzing the chemical components, including various metabolites used in traditional Chinese medicine^59^. Here, the metabolomes of *E. japonica* leaves, flowers, and roots were determined using a widely targeted metabolomic analysis method based on liquid chromatography and tandem mass spectrometry (LC-MS/MS), as described by Chen et al.^60^, and many additional metabolites, including phenols, terpenoids, and alkaloids, were detected. These results supplement available information on the medicinally valuable biochemical substances present in *E. japonica*. These data were also used to identify and annotate some of the genes encoding key biosynthetic enzymes related to phenol, terpenoid, and polysaccharide biosynthesis in *E. japonica*. The data allowed the further evaluation of the evolutionary relationships among these genes. Taken together, these results provide valuable insights into the molecular mechanisms and genetic background facilitating the production of several important medicinal compounds in this species and lay a foundation for conducting further studies on the molecular pharmacology of *E. japonica*.

## Conclusion

This study produced a high-quality draft genome of yellow-fleshed *E. japonica* and high-throughput metabolomes of its leaf, flower, and root tissues. A total of 45,492 putative protein-encoding genes and 577 metabolites were identified. In addition, 91 phenols, 81 flavonoids, 28 terpenoids, and some saccharide metabolites with potential health benefits and genes related to the biosynthesis of these metabolites were highlighted. Overall, this study describes a high-quality draft genome and provides a global view of the fundamental molecular components that contribute to the medicinal value of *E. japonica*.

## Materials and methods

### Cultivar description and genome materials

The ‘Big Five-pointed Star’ cultivar was subjected to sequencing to assemble a draft genome, which was then used as a reference genome for the SNP mapping of resequencing data. The ‘Big Five-pointed Star’ drupe has excellent characteristics, including a high average single fruit weight, high edible rate, high soluble solid contents, juiciness and delicious flavor. As a result, the Big Five-pointed Star has become a popular cultivar, with the most rapid development and expansive planting area in China^47^. Descriptions of the flesh color and geographical origins of the 53 *E. japonica* cultivars used for GWAS are provided in Table S15.

### DNA extraction

Total DNA was extracted from the young leaves of ‘Big Five-pointed Star’ using a modified protocol based on the CTAB method^61^ and was then treated with RNase (Thermo Fisher Scientific, Waltham, MA, United States) at 37 °C for 1 h. The quality and concentration of the total extracted genomic DNA were determined using agarose gel electrophoresis and ND-1000 spectrophotometry (NanoDrop Technologies Inc., Wilmington, DE, USA).

### Illumina short-read library construction and sequencing

The Illumina paired-end library (350 bp) was constructed according to the manufacturer’s instructions via the following steps: qualified DNA was fragmented, and segments of ~350 bp in length were selected on a 3% agarose gel for further analysis. End repair and A-tailing were performed, and Illumina-compatible adaptors were added to the selected DNA fragments before PCR amplification using Illumina adapter-specific primers, which completed paired-end sequencing library construction. Raw short-read sequences of ~150 bp in length were generated on the Illumina HiSeq 4000 platform (Illumina Inc.).

### Survey of the ‘Big Five-pointed Star’ genome based on short-read data by *k*-mer analysis

A *k*-mer analysis was performed using the K-mer Analysis Toolkit (KAT) program^62^ to determine an initial estimate based on genome size, heterozygosity, and the repetitive rate of the ‘Big Five-pointed Star’ genome. The following formula was used: genome size = (total nucleotide number)/(average sequencing depth) = (total number of *k*-mers)/(average *k*-mer depth). The K value used the maximum number of odd numbers that met the following criteria: 4^K/genome >200.

### PacBio long-read library construction, sequencing, and raw data statistics

The long-read sequencing library (20 kb) was constructed according to the PacBio guidelines and was completed as follows: G-tube fragmentation of genomic DNA, damage repair of fragmented DNA, end-repair of fragmented DNA, ligation of fragmented DNA with dumbbell-shaped adaptors, digestion of DNA segments using exonuclease, and selection of target segments using BluePippin. Long-read sequences were generated on the PacBio sequencing platform (Pacific Biosciences Inc., California, USA).

### Assembly and evaluation of the integrity of the draft genome

To assemble the draft genome of *E. japonica* using the long-read sequencing data, subreads with low quality (<Q20) and short lengths (<500 bp) were removed, and the remaining subreads were corrected using Canu software^63^. The corrected data were then assembled into a draft genome sequence by using WTDBG (https://hpc.ilri.cgiar.org/wtdbg2-software) with parameters ‘-p 19 -AS 2’ (wtdbg, RRID:SCR_017225), Falcon software^64^ with the parameters set to the defaults, and Canu software with the parameters set to canu: errorRate 0.045. The results of these three analyses were then optimized using the Quickmerge ideology^65^ under its default parameters and were improved by correcting errors by combining the short-read data using Pilon software^66^ with default parameter settings.

The following three methods were then used to evaluate the completeness of the draft genome. The first consisted of BLAST searches of the assembled draft genome with a standard of more than 70% identity against the CEGMA database^24^, which included 458 core eukaryotic genes (CEGs) and 248 highly conserved CEGs. The second consisted of BLAST searches of the assembled draft genome with at least 70% identity against the embryophyta_odb10 dataset in the BUSCO v4.0 database (https://busco.ezlab.org/busco_v4_data.html), which included 1440 conserved core plant genes. Finally, the short-read sequencing data were mapped to the assembled draft genome using BWA software^67^ (v0.7.10-r789; aln model; other parameters were set to default).

### Hi-C sequencing library construction

A Hi-C sequencing library was constructed according to protocols described by Servant et al.^68^ and Burton et al.^69^. Briefly, the cells of young leaves of the ‘Big Five-pointed Star’ cultivar were fixed with formaldehyde and then dissociated, and the cross-linked products were treated with restriction endonucleases to produce cohesive ends. A biotin marker was introduced at the cohesive ends, which were repaired to produce blunt ends. The blunt ends were ligated; the cross-links were released to separate DNA from proteins; the DNA was extracted; a Covaris E220 instrument (Covaris, Brighton, UK) was used to fragment DNA to the correct size and then repair the ends; the fragmented DNA segments were purified by gel electrophoresis and recycled with a QIAquick Gel Extraction Kit (Qiagen Inc., Germany); those DNA segments without a biotin marker were removed; Poly(A) sequences were added to the remaining DNA segments including biotin markers; PCR adaptors were added; PCR was performed; and the PCR products were purified by gel electrophoresis and recycled by using a QIAquick Gel Extraction Kit (Qiagen Inc.).

### Hi-C sequencing and assembly

The Illumina HiSeq 4000 (Illumina, San Diego, CA, USA) sequencing platform was used for paired sequencing by synthesis. The paired reads were mapped to the assembled *E. japonica* draft genome using the BWA program (v0.7.10-r789; aln model; other parameters were set to default)^67^. LACHESIS software^70^ was used to scaffold the contigs onto the chromosomes using the following parameters: CLUSTER_MIN_RE_SITES = 53; CLUSTER_MAX_LINK_DENSITY = 2; CLUSTER_NONINFORMATIVE_RATIO = 2; ORDER_MIN_N_RES_IN_TRUN = 21; ORDER_MIN_N_RES_IN_SHREDS = 22.

### Repetitive sequence prediction and annotation

A unique database for identifying repetitive sequences in the genome was constructed with the help of LTR_FINDER v1.05^71^, MITE-Hunter^72^, RepeatScout v1.0.5 (Price et al.)^73^, and PILER-DF v2.4 software^74^ based on structure and de novo prediction. This unique database was then merged with the Repbase database^75^ to generate the final repetitive sequence database, and PASTEClassifier software^76^ was used to classify the database. Finally, Repeatmasker v4.0.6 software^77^ was used to predict repetitive sequences in the draft genome based on a well-constructed, repeating sequence database.

### Protein-coding gene prediction and functional annotation

Protein-coding genes based on nonrepetitive sequences in the draft genome were predicted using three methods: (1) De novo prediction (Ab initio) using Genscan software^78^, Augustus v2.4^79^, GlimmerHMM v3.0.4^80^, GeneID v1.4^81^, and SNAP^82^; (2) homologous species prediction with GeMoMa v1.3.1^83^ software (based on gene sequences of *A. thaliana*, *Oryza sativa japonica*, *M. domestica*, *P. bretschneideri*, and *F. vesca* from gene and expression databases of NCBI (https://www.ncbi.nlm.nih.gov/guide/genes-expression/); and (3) unigene and EST prediction with TransDecoder v2.0.1 (http://transdecoder.github.io/ [last accessed May 8, 2019]) and GeneMarkS-T v5.1^84^ software based on 150 228 *E. japonica* unigenes assembled from the transcriptomic data with reference transcripts based on *E. japonica* expressed sequence tags collected from the NCBI dbEST database (http://www.ncbi.nlm.nih.gov/dbEST/) using HISAT v2.0.4^85^ and StringTie v1.2.3^86^ software. In addition, PASA v2.4.1 software (https://github.com/PASApipeline/PASApipeline/releases) was used based on the *E. japonica* unigenes from the transcriptome data with nonreferenced transcripts.

The results of the above three methods were integrated using EVidenceModeler (EVM) v1.1.1 software (https://github.com/EVidenceModeler/EVidenceModeler/releases/tag/v1.1.1). In addition, five databases, including the nonredundant protein sequence database (Nr) (https://www.ncbi.nlm.nih.gov/refseq/), UniProtkb/SwissProt protein knowledgebase database (TrEMBL) (https://www.uniprot.org/statistics/TrEMBL), GO database (http://geneontology.org/), Eukaryotic Orthologous Groups database (KOG) (http://www.ncbi.nlm.nih.gov/COG/), and KEGG database (https://www.genome.jp/kegg/), were used to annotate the molecular functions of these predicted protein-coding genes using BLAST v2.10.0 software (https://www.ncbi.nlm.nih.gov/books/NBK131777/.) with a threshold e-value of 1e−5.

### RNA gene prediction and annotation

Infernal 1.1 software^87^ was used to predict rRNA genes based on Rfam (https://rfam.xfam.org/) and miRNAs based on the miRBase database (http://www.mirbase.org/). The tRNAscan-SE v1.3.1 program^88^ was used to identify tRNA loci. The tRNAscan-SE v1.3.1 program^88^ was used to identify tRNA loci. GenBlastA v1.0.4 software^89^ was used to search homologous RNA gene sequences via BLAST searches of the remaining draft genome regions after shielding the predicted gene sequences. Pseudogenes were identified by searching for immature termination codons and shift code mutations using GeneWise v2.4.1 software^90^.

### Data resource and processing for comparative genomics

Protein-coding gene sets from apple (https://www.rosaceae.org/species/malus/malus_x_domestica/genome_v3.0.a1), peach (https://www.rosaceae.org/species/prunus_persica/genome_v2.0.a1), pear (https://www.rosaceae.org/species/pyrus_bretschneideri/genome_v1.1), sweet cherry (https://www.rosaceae.org/species/prunus_avium/genome_v1.0.a1), Chinese plum, black raspberry (https://www.rosaceae.org/analysis/268), woodland strawberry (https://www.rosaceae.org/species/fragaria/fragaria_vesca/genome_v1.0), rose (https://www.rosaceae.org/analysis/288), and *A. thaliana* (https://www.arabidopsis.org/) were used to perform a comparative genomics analysis of *E. japonica*.

### Identification of gene families

The gene sets of the above species and *E. japonica* were aligned using all-against-all Blastp^91^ according to e-values ≤ 1e5 and ≤500 hits. OrthoFinder v2.3.7 software^92^ was used for the gene family classification of the protein sequences of the ten species, and the ‘Protein Analysis Through Evolutionary Relationships’ (PANTHER v15) database (http://pantherdb.org/) was used for the annotation of the obtained gene families. Finally, GO and KEGG enrichment analyses were performed by using clusterProfile v3.14.0 software^93^.

### Phylogenetic tree construction

The protein sequences of 594 single-copy genes were used to construct an evolutionary tree using IQ-TREE v1.6.11 software^94^. Specifically, MAFFT v7.205 software^95^ was used to align the sequences, and Gblocks v0.91b^96^ was used to remove regions with poor sequence alignment or significant differences using the following parameters: -b5 = H. ModelFinder^97^ was used for model detection, and the best-obtained model was JTT + F + G4, which was then used to construct a maximum likelihood (ML) evolutionary tree with the number of bootstraps set to 1000 and *A. thaliana* as the outgroup. The MCMCTree module of PAML v4.9i software^98^ was used to calculate the divergence times between species. Finally, the evolutionary tree with divergence times was graphically presented using MCMCTreeR^99^.

### Identification and enrichment analysis of single-copy genes under positive selection

The modular CodeML built-in PAML v4.9d package^98^ was used to detect the selection pressure on specific single-copy genes in each of the nine species based on the construction of phylogenetic trees. Single-copy orthologs with nonsynonymous/synonymous (D_N_/D_S_) substitution ratios >1 (*p* ≤ 0.05 by chi-square test) were indicated to be under positive selection (rapid evolution or adaptive evolution). These genes were then subjected to GO and KEGG enrichment using GOseq^100^ and KOBAS^101^ software, respectively.

### Gene family expansion and contraction and functional enrichment analysis

The expansion and contraction of the gene families of *E. japonica* and those of eight other Rosaceae species were analyzed using CAFÉ software^102^ based on the phylogenetic evolutionary tree. Significant expansion or contraction was indicated by *p* < 0.05. GO and KEGG enrichment analyses were performed on the genes in the expanded families using GOseq software^100^ and KOBAS^101^ software, respectively.

### GWAS

The resequencing data of the 52 *E. japonica* cultivars and the methods for SNP dataset detection have been previously reported^103^. The EMMAX^30^ and Fastlmm^31^ programs, with a compressed mixed linear model and linear mixed model, respectively, were used to perform the GWAS based on the original SNP dataset filtered according to a threshold minor allele frequency <0.05 and locus integrity >0.8. Manhattan and quantile-quantile plots were constructed using the R package (https://rstudio.com/products/rpackages/).

### Sampling, sample preparation, and metabolite extraction

A total of nine samples (three from leaves, three from flowers, and three from roots) of the ‘Big Five-pointed Star’ cultivar were collected at different developmental stages and subjected to metabolome analysis. The freeze-dried samples were crushed using an MM 400 mixer mill (Retsch, Haan, Germany) and zirconia beads for 1.5 min at 30 Hz. Then, 100 mg of each sample was subjected to 70% methanol extraction overnight at 4 °C. After centrifugation at 10,000 × *g* for 10 min, the extracts were absorbed (CNWBOND Carbon-GCB SPE Cartridge, 250 mg, 3 mL; ANPEL Laboratory Technologies, Shanghai, China) and filtered (SCAA-104, 0.22 μm pore size; ANPEL) before being subjected to UPLC-MS/MS analysis.

### UPLC conditions

Sample extracts were analyzed using a UPLC-ESI-MS/MS system (UPLC: Shim-pack UFLC CBM30A system, Shimadzu, Kyoto, Japan; MS: Applied Biosystems 4500 QTRAP, AB Sciex, Framingham, MA, USA). The analytical conditions were as follows: Agilent SB-C18 UPLC column (1.8 µm, 2.1 mm × 100 mm; Agilent Technologies, Santa Clara, CA, USA) and a mobile phase comprising solvent A (pure water with 0.1% formic acid) and solvent B (acetonitrile). Sample measurements were performed using a gradient program that started with 95% A and 5% B. Within 9 min, a linear gradient with an endpoint of 5% A and 95% B was programmed, and the composition of 5% A and 95% B was maintained for 1 min. This was then reversed to 95% A and 5% B within 1.10 min, which was maintained for 2.9 min. The column oven temperature was set at 1–40 °C, and the injection volume was 4 μL. The effluent was connected to ESI-triple quadrupole linear ion trap (QTRAP)-MS.

### ESI-Q TRAP-MS/MS

LIT and triple quadrupole scans were acquired using a triple quadrupole-linear ion trap mass spectrometer (QTRAP; API 4500 QTRAP UPLC/MS/MS System) equipped with an ESI Turbo Ion-Spray interface, operating in both positive and negative ion modes and were controlled using Analyst Software 1.6.3 (https://sciex.com/products/software/analyst-software; AB Sciex). The ESI source operation parameters were as follows: ion source, turbo spray; source temperature, 550 °C; ion spray voltage: 5500 V (positive ion mode)/-4500 V (negative ion mode); and ion source gas I (GSI), gas II (GSII), and curtain gas (CUR) values set at 50, 60, and 30 psi, respectively. Instrument tuning and mass calibration were performed using 10 and 100 μmol/L polypropylene glycol solutions in triple quadrupole and LIT modes, respectively. Triple quadrupole scans were acquired after the completion of a multiple reaction monitoring (MRM) experiment with the collision gas (nitrogen) set to 5 psi. The declustering potential and collision energy of individual MRM transitions were then determined before further declustering potential and collision energy optimization. A specific set of MRM transitions was monitored in each period according to the metabolites eluted within that period^60^.

### Qualitative and quantitative analysis of metabolites

The qualitative analysis of metabolites was performed using secondary spectrum information with reference to the MetWare database (Maiwei Metabolism, Wuhan, China). Furthermore, repeat signals, including those from the K ^+ ^, Na^+^, and NH_4 _^+ ^ions, as well as repeated signals from fragmented ions, indicating a high molecular weight substance, were removed. Then, metabolites were quantified via the MRM mode analysis of triple quadrupole mass spectrometry data^104^.

### MS data analysis

The mass spectrum data were processed using Analyst Software 1.6.3 (https://sciex.com/products/software/analyst-software), and samples from the same organ were treated as repeats. Principal component analysis, Pearson’s correlation analysis, differential expression analysis, and heat map generation were all performed using the statistical module in R (version 3.1.1) (https://www.r-project.org/). The KEGG database (https://www.kegg.jp/) was used to annotate and elucidate the biosynthetic pathways of different metabolites.

## Supplementary information

Supplementary tables

Figure S1

Figure S2

Figure S3

Figure S4

Figure S5

Figure S6

Figure S7

Figure S8

Figure S9

Figure S10

Figure S11

Figure S12

Figure S13

Figure S14

Figure S15

Figure S16

Figure S17

Figure S18

Figure S19

Supplementary data file 1

Supplementary data file 2

Supplementary data file 3

Supplementary data file 4

Supplementary data file 5

Supplementary data file 6

Supplementary data file 7

Supplementary data file 8

Supplementary data file 9

Supplementary data file 10

Supplementary data file 11

Supplementary data file 12

Supplementary data file 13

Supplementary data file 14

Supplementary data file 15

Supplementary data file 16

## Data Availability

The draft genome sequence in FASTA format and protein-coding gene sequences in GFF3 format were deposited in the China National Center for Bioinformation Database https://bigd.big.ac.cn/gsub/: accessible ID: GWHAOTB00000000.
