# Supplementary material for: A draft genome, resequencing, and metabolomes reveal the genetic background and molecular basis of the nutritional and medicinal properties of loquat (Eriobotrya japonica (Thunb.) Lindl)
Source: Hortic Res. 2021 Nov 1;8:231. doi: 10.1038/s41438-021-00657-1 (PMC8558328; doi:10.1038/s41438-021-00657-1)
Supplement: Supplementary file 1 — Supplementary tables [file 41438_2021_657_MOESM1_ESM.docx]

Supplementary Information

**Figure legends**

**Figure S1** Genome survey based on the frequency distribution of k-mers (k = 21) of *Eriobotrya japonica* genome-based short-reads

Note: A total of 44,651,396,663 K-mers were identified, and the peak of K-mer depth observed was 57.1. Genome size can be estimated as (total K-mer number)/(the volume peak). Therefore, the genome size of loquat was evaluated as 749.25 Mb.

**Figure S2** Length distribution of sub-reads by Pacbio sequencing

**Figure S3** Hi-C assembled based on contigs from long-reads

Note: Hi-C map showing genome-wide all-by-all interactions of the *E. japonica* genome and a high resolution of each of the 17 chromosomes scaffolded and assembled independently.

**Figure S4** Loquat specific family genes and their (a) GO and (b) KEGG enrichment statistics.

**Figure S**5 Loquat single-copy families genes undergone a positive selection and their (a) GO and (b) KEGG enrichment statistics.

**Figure S6** Loquat expansion family genes and their GO and KEGG enrichment statistics.

**Figure S7** QQ-plot of GWAS by Emmax (a) and Fastlmm (b)

**Figure S8** Principal component analysis based on metabolites of all samples

**Figure S9** Pearson`s correlation based on metabolites of all samples

**Figure S10** Metabolites involved in the metabolism pathway of phenylpropanoids biosynthesis.

Note: The colored solid dots represent a match annotated by loquat metabolites

**Figure S11** Genes involved in the metabolism pathway of Phenylpropanoids biosynthesis.

Note: The yellow box represents a match annotated by a loquat gene.

**Figure S12** Metabolites involved in the metabolism pathway of flavonoid biosynthesis.

Note: The colored solid dots represent a match annotated by loquat metabolites

**Figure S13** Genes involved in the metabolism pathway of flavonoid biosynthesis.

Note: The yellow box represents a match annotated by a loquat gene.

**Figure S14** Genes involved in the metabolism pathway of terpenoid backbone biosynthesis.

Note: The yellow box represents a match annotated by a loquat gene.

**Figure S15** Genes involved in the metabolism pathway of monoterpenoid biosynthesis.

Note: The yellow box represents a match annotated by a loquat gene.

**Figure S16** Genes involved in the metabolism pathway of diterpenoid biosynthesis. Note: The yellow box represents a match annotated by a loquat gene.

**Figure S17** Genes involved in the metabolism pathway of sesquiterpenoid and triterpenoid biosynthesis

Note: The yellow box represents a match annotated by a loquat gene

**Figure S18** Genes involved in the metabolism pathway of galactose metabolism

Note: The yellow box represents a match annotated by a loquat gene

**Figure S19** Genes involved in the metabolism pathway of fructose and mannose metabolism

Note: The yellow box represents a match annotated by a loquat gene

**Supplementary tables**

Table S1 Statistics for short-read sequencing data generated by Illumina Hiseq 4000.

| Items | Counts |
| --- | --- |
| Total reads | 688,175,216 |
| Data (Gb) | 51.54 |
| Depth (×) | 68.79 |
| Q20 (%) | 98.33 |
| Q30 (%) | 94.98 |

Table S2 Summary of Big Five-pointed Star genome by K-mer analysis

| Items | Counts |
| --- | --- |
| Tatal Number of K-mer | 44,651,396,663 |
| Number of K-mer with abnormal depth | 1,890,484,787 |
| Number of K-mer with normal depth | 42,760,911,876 |
| Mean of K-mer depth | 57.1(57) |
| Genome size (Total K-mer/Mean of K-mer depth) | 749.25 (42760911876/57) |
| Percentage of K-mer with depth more than 114.2 | 53.40% |
| Percentage of K-mer with depth of about 28.5 | 0.31% |
| Percentage of GC content | 38.58% |

Table S3 Statistics of long read sequencing data generated by Pacbio platform

| Subreads | Counts |
| --- | --- |
| Number | 6,028,317 |
| Total Bases (bp) | 36,897,900,452 |
| Reads N50 (bp) | 11,469 |
| Mean Length (bp) | 6,121 |
| Longest Read (bp) | 87,832 |

Table S4 The initial assembled information of draft genome at contig level

| Assembly software | Contig number | Contig length (bp) | Contig N50 (bp) | Contig N90 (bp) | Contig Max (bp) | GC content (%) |
| --- | --- | --- | --- | --- | --- | --- |
| WTDBG | 17,199 | 611,343,215 | 53,291 | 17,582 | 424,650 | 39.06 |
| Falcon | 7,188 | 316,939,488 | 80,646 | 21,413 | 718,029 | 38.03 |
| Canu | 5,248 | 739,768,422 | 259,183 | 56,015 | 2,558,044 | 37.95 |
| Canu+WTDBG | 3677 | 733,320,379 | 588,263 | 66,800 | 4,966,828 | 37.89 |

Note: Contig Number, Contig length (bp), Contig N50 (bp) and Contig N90 only accounted these Contigs with length more than 1000bp; Contig max (bp): the longest contig.

Table S5 Integrity estimation of initial assembled draft genome

| Database | Aligning dataset | Counting (%) |
| --- | --- | --- |
| CEGMA v2.5 | 458 CEGs* present in assembly (%) | 447 (97.60) |
|  | 248 highly conserved CEGs present (%) | 238 (95.79%_ |
| embryophyta_odb9 database | 2,326 Complete BUSCOs (%) | 2,170 (93.29%) |
|  | Complete and single-copy BUSCOs (%) | 1,450 (62.34%) |
|  | Complete and duplicated BUSCOs (%) | 689 (29.62%) |
|  | Fragmented BUSCOs (%) | 31 (1.33%) |
|  | Missing BUSCOs (%) | 156 (6.71%) |
| Short read data | Total reads | 336,487,099 |
|  | Mapped reads (%) | 326,897,216 ( 97.15%) |
|  | Properly mapped reads (%) | 322,784,522 (93.81%) |

Table S6 Summary of Hi-C clean data aligned with intial assembled draft genome

| Library | Mapping type | Number | Ratio (%) |
| --- | --- | --- | --- |
| H01 | Total read pairs | 305,977,336 | 100 |
|  | Mapped reads | 498,488,714 | 81.46 |
|  | Unique mapped read pairs | 151,920,188 | 49.65 |
|  | Valid Interaction Pairs | 70,979,954 | 46.72 |
|  | Dangling End Pairs | 67,267,873 | 44.28 |
|  | Re-ligation Pairs | 2,064,369 | 1.36 |
|  | Self-cycle Pairs | 822,001 | 0.54 |
|  | Dumped Pairs | 10,785,991 | 7.1 |
| H02 | Total read pairs | 15,217,613 | 100 |
|  | Mapped reads | 25,284,337 | 83.08 |
|  | Unique mapped read pairs | 7,785,104 | 51.16 |
|  | Valid Interaction Pairs | 3,668,159 | 47.12 |
|  | Dangling End Pairs | 3,367,679 | 43.26 |
|  | Re-ligation Pairs | 104,537 | 1.34 |
|  | Self-cycle Pairs | 42,526 | 0.55 |
|  | Dumped Pairs | 602,203 | 7.74 |
| Total | Total read pairs | 321,194,613 | 100 |
|  | Mapped reads | 523,773,337 | 81.54 |
|  | Unique mapped read pairs | 159,705,292 | 49.72 |
|  | Valid Interaction Pairs | 74,648,113 | 46.74 |
|  | Dangling End Pairs | 70,635,552 | 44.23 |
|  | Re-ligation Pairs | 2,168,537 | 1.36 |
|  | Self-cycle Pairs | 864,526 | 0.54 |
|  | Dumped Pairs | 11,388,194 | 7.13 |

Table S7 Statistics of Hi-C assembly

| Group | Sequence Number | Sequence Length (bp) |
| --- | --- | --- |
| LG01 | 319 | 54,973,903 |
| LG02 | 182 | 48,533,986 |
| LG03 | 353 | 48,449,659 |
| LG04 | 284 | 47,596,596 |
| LG05 | 258 | 45,516,778 |
| LG06 | 163 | 44,160,895 |
| LG07 | 229 | 43,594,283 |
| LG08 | 184 | 43,590,855 |
| LG09 | 255 | 42,209,654 |
| LG10 | 189 | 41,587,509 |
| LG11 | 214 | 40,593,040 |
| LG12 | 244 | 39,610,895 |
| LG13 | 225 | 38,544,535 |
| LG14 | 170 | 37,931,386 |
| LG15 | 113 | 37,910,185 |
| LG16 | 221 | 37,836,838 |
| LG17 | 122 | 34,759,910 |
| Total Sequences Clustered (%) | 3,725 (94.59%) | 727,400,907(99.19%) |
| Total Sequences Ordered and Oriented (%) | 2,181 (58.38%) | 644,881,393(87.94%) |
| Total contigs | 3,938 | 733,320,379 |

Table S8 Statistics of repetitive sequence

| Type | Number | Length | Rate (%) |
| --- | --- | --- | --- |
| ClassI | 754,305 | 423,602,158 | 57.76 |
| ClassI/DIRS | 36,518 | 32,038,908 | 4.37 |
| ClassI/LINE | 45,374 | 13,572,449 | 1.85 |
| ClassI/LTR | 89,595 | 32,901,447 | 4.49 |
| ClassI/LTR/Copia | 231,395 | 158,900,019 | 21.67 |
| ClassI/LTR/Gypsy | 240,193 | 204,741,860 | 27.92 |
| ClassI/PLE\|LARD | 107,570 | 25,544,538 | 3.48 |
| ClassI/SINE | 1,161 | 234,501 | 0.03 |
| ClassI/SINE\|TRIM | 43 | 17,307 | 0 |
| ClassI/TRIM | 2,315 | 2,155,692 | 0.29 |
| ClassI/Unknown | 141 | 32,170 | 0 |
| ClassII | 389,888 | 113,401,913 | 15.46 |
| ClassII/Crypton | 4 | 214 | 0 |
| ClassII/Helitron | 65,650 | 16,946,497 | 2.31 |
| ClassII/MITE | 3,071 | 681,787 | 0.09 |
| ClassII/Maverick | 1,521 | 278,758 | 0.04 |
| ClassII/TIR | 272,594 | 88,267,148 | 12.04 |
| ClassII/Unknown | 47,048 | 10,755,699 | 1.47 |
| Potential Host Gene | 5,047 | 1,220,005 | 0.17 |
| Total | 1,149,934 | 516,110,047 | 70.38 |

Table S9 Statistics of predicting genes

| Method | Software | Species | Gene number |
| --- | --- | --- | --- |
| Ab initio | Genscan | - | 30,827 |
|  | Augustus | - | 50,606 |
|  | GlimmerHMM | - | 39,724 |
|  | GeneID | - | 52,963 |
|  | SNAP | - | 47,857 |
| Homology-based | GeMoMa | Arabidopsis_thaliana | 34,984 |
|  |  | Oryza_sativa_Japonica | 36,141 |
|  |  | Malus_domestica | 51,914 |
|  |  | Pyrus_bretschneideri | 43,701 |
|  |  | Fragaria_vesca | 40,767 |
| RNAseq | PASA | - | 42,316 |
|  | GeneMarkS-T | - | 36,467 |
|  | TransDecoder | - | 62,567 |
| Integration | EVM | - | 45492 |

Table S10 Statistics of prodicted protein-coding genes information

| Items | Counts |
| --- | --- |
| Total Gene Number | 45,492 |
| Total Gene Length (bp) | 155,499,470 |
| Average Gene Lengh (bp) | 3,418 |
| Total Exon Length (bp) | 69,617,871 |
| Average Exon Length (bp) | 1,530 |
| Total Intron Length (bp) | 85,881,599 |
| Average Intron Length (bp) | 1,888 |

Table S11 Annotation statistics of predicted protein coding genes

| Database | Annotated number (%) |
| --- | --- |
| GO | 21,415(47.07%) |
| KEGG | 14,084(30.96%) |
| KOG | 25,543(56.15%) |
| TrEMBL | 44,411(97.62%) |
| Nr | 45,078(99.09%) |
| Combinated | 45,090(99.12%) |

Table S12 Statistics of predicted non-coding RNA genes and pseudogenes in draft genome

| RNA classification | Gene Number | Family Number |
| --- | --- | --- |
| rRNA | 10,426 | 4 |
| tRNA | 691 | 24 |
| miRNA | 165 | 25 |
| Pseudogene | 8,314 |  |

Table S13 The number of SNPs and genes detected by genome-wide association study of loquat flesh color

| Coputational model | P-value | P-value | Number SNP of signloci | Number genes of signloci |
| --- | --- | --- | --- | --- |
| Color of Emmax | 5.514e-08_100 | <0.005 | 4 | 72 |
|  | 5.514e-09_100 | <0.001 | 1 | 16 |
| Color of Fastlmm | 5.532e-08_100 | <0.005 | 91 | 571 |
|  | 5.532e-09_100 | <0.001 | 22 | 232 |

Signloci: Loci be significant association with flesh color of loquat

Table S14 Chromosome alignment results of draft genome between ‘Big Five-pointed Star’ and ‘Seven Star’

| Big Five-pointed Star | Seven Star | Orientation |
| --- | --- | --- |
| LG10 | GWHAAZU00000010 | - |
| LG11 | GWHAAZU00000012 | - |
| LG12 | GWHAAZU00000013 | - |
| LG13 | GWHAAZU00000017 | - |
| LG14 | GWHAAZU00000011 | - |
| LG15 | GWHAAZU00000014 | - |
| LG16 | GWHAAZU00000016 | - |
| LG17 | GWHAAZU00000015 | + |
| LG1 | GWHAAZU0000001 | - |
| LG2 | GWHAAZU0000003 | + |
| LG3 | GWHAAZU0000007 | - |
| LG4 | GWHAAZU0000002 | - |
| LG5 | GWHAAZU0000004 | - |
| LG6 | GWHAAZU0000008 | - |
| LG7 | GWHAAZU0000009 | - |

Note: + represents the same haplotype of assembled chromosomes; - represents the complementary haplotype of assembled chromosomes. This alignment was executed the software Mummer 4 (https://github.com/mummer4/mummer)

Table S15 Breeding information of loquat cultivars used for genome-wide association study

| Code | Sample name | Flesh color | Name of cultivars | Breeding origin |
| --- | --- | --- | --- | --- |
| C01 | C_BL | White | Baili | Fujian |
| C02 | C_JFB | White | Jiefangbai | Fujian |
| C03 | C_JXB | White | Jinxingbai | Fujian |
| C04 | C_CB | White | Changbai | Fujian |
| C05 | C_WGB | White | Wugongbai | Fujian |
| C06 | C_JCB | White | Jingchengbai | Fujian |
| C07 | C_RTB | White | Ruantiaobai | Zhejiang |
| C08 | C_NHB | White | Ninghaibai | Zhejiang |
| C09 | C_BY | White | Baiyu | Jiangsu |
| C10 | C_JDB | White | Jidanbai | Jiangsu |
| C11 | C_XBS | White | Xiaobaisha | Jiangsu |
| C12 | C_GY | White | Guanyu | Jiangsu |
| C13 | C_BMM | White | White Mogi | Japan |
| C14 | C_MHH | Yellow | Maihouhuang | Shaanxi |
| C15 | C_BH | Yellow | Bahong | Jiangsu |
| C16 | C_CL5 | Yellow | Changlv5 | Jiangsu |
| C17 | C_TZ | white | Tianzhong | Jiangsu |
| C18 | C_BZ | Yellow | Baozhu | Jiangsu |
| C19 | C_TP | Yellow | Tongpi | Jiangsu |
| C20 | C_PJZ | white | Pijizhong | Jiangsu |
| C21 | C_CN | Yellow | Chuannao | Jiangsu |
| C22 | C_CL4 | Yellow | Chuanglv No.4 | Jiangsu |
| C23 | C_GLJ | Yellow | Gaoliangjiang | Jiangsu |
| C24 | C_BTZ | white | Bingtangzhong | Jiangsu |
| C25 | C_QZ | Yellow | Qingzhong | jiangsu |
| C26 | C_HB3 | Yellow | Huabao No.3 | Hubei |
| C27 | C_HB2 | Yellow | Huabao No.2 | Hubei |
| C28 | C_SJ | Yellow | Sijie | Yunnan |
| C29 | C_GR | Yellow | Guangrong | Anhui |
| C30 | C_LQ | Yellow | Longquan No.1 | Sichuan |
| C31 | C_DWX | Yellow | Dawuxing | Sichuan |
| C32 | C_WZ | Yellow | Wanzhong | Fujian |
| C33 | C_ZS | Yellow | Zaoseng | Fujian |
| C34 | C_MHX | Yellow | Meihuaxia | Fujian |
| C35 | C_DZ | Yellow | Dazhong | Fujian |
| C36 | C_JFZ | Yellow | Jiefangzhong | Fujian |
| C37 | C_ZZ6 | Yellow | Zaozhong No.6 | Fujian |
| C38 | C_CH3 | Yellow | Changhong No3 | Fujian |
| C39 | C_DHP | Yellow | Dahongpao | Zhejiang |
| C40 | C_XZ | Yellow | Xiangzhong | Zhejiang |
| C41 | C_XLR | Yellow | Xilinri | Zhejiang |
| C42 | C_XHR | Yellow | Xinhuangrou | Zhejiang |
| C43 | C_SM | Yellow | Suangming | Zhejiang |
| C44 | C_MJ2 | Yellow | Mojia2 | Guangdong |
| C45 | C_MJ | Yellow | Maojia | Guangdong |
| C46 | C_Cri | Yellow | Cri | Italy |
| C47 | C_IDL | Yellow | Italio | Italy |
| C48 | C_JL | Yellow | Javierin | Spain |
| C49 | C_MK | Yellow | Marc | Spain |
| C50 | C_WLL | Yellow | Ullera | Spain |
| C51 | C_BCM | Yellow | BCM | Spain |
| C52 | C_MM | Yellow | Mogi | Japan |
| C53 | C_HJK | Yellow | Golden Nugget | American |
