# Supplementary figures and images for: A draft genome, resequencing, and metabolomes reveal the genetic background and molecular basis of the nutritional and medicinal properties of loquat (Eriobotrya japonica (Thunb.) Lindl)
Source: Hortic Res. 2021 Nov 1;8:231. doi: 10.1038/s41438-021-00657-1 (PMC8558328; doi:10.1038/s41438-021-00657-1)

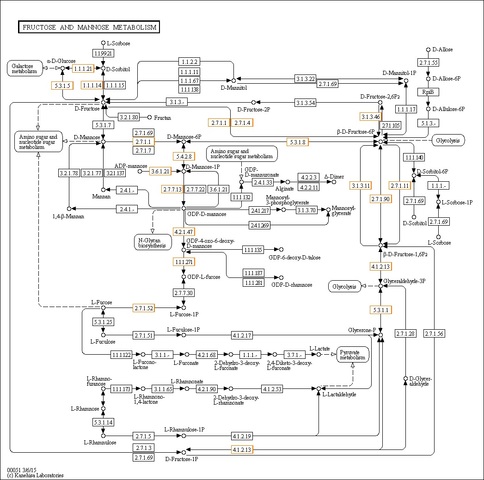

Supplement: Supplementary file 2 — Figure S1 [file 41438_2021_657_MOESM2_ESM.jpg]

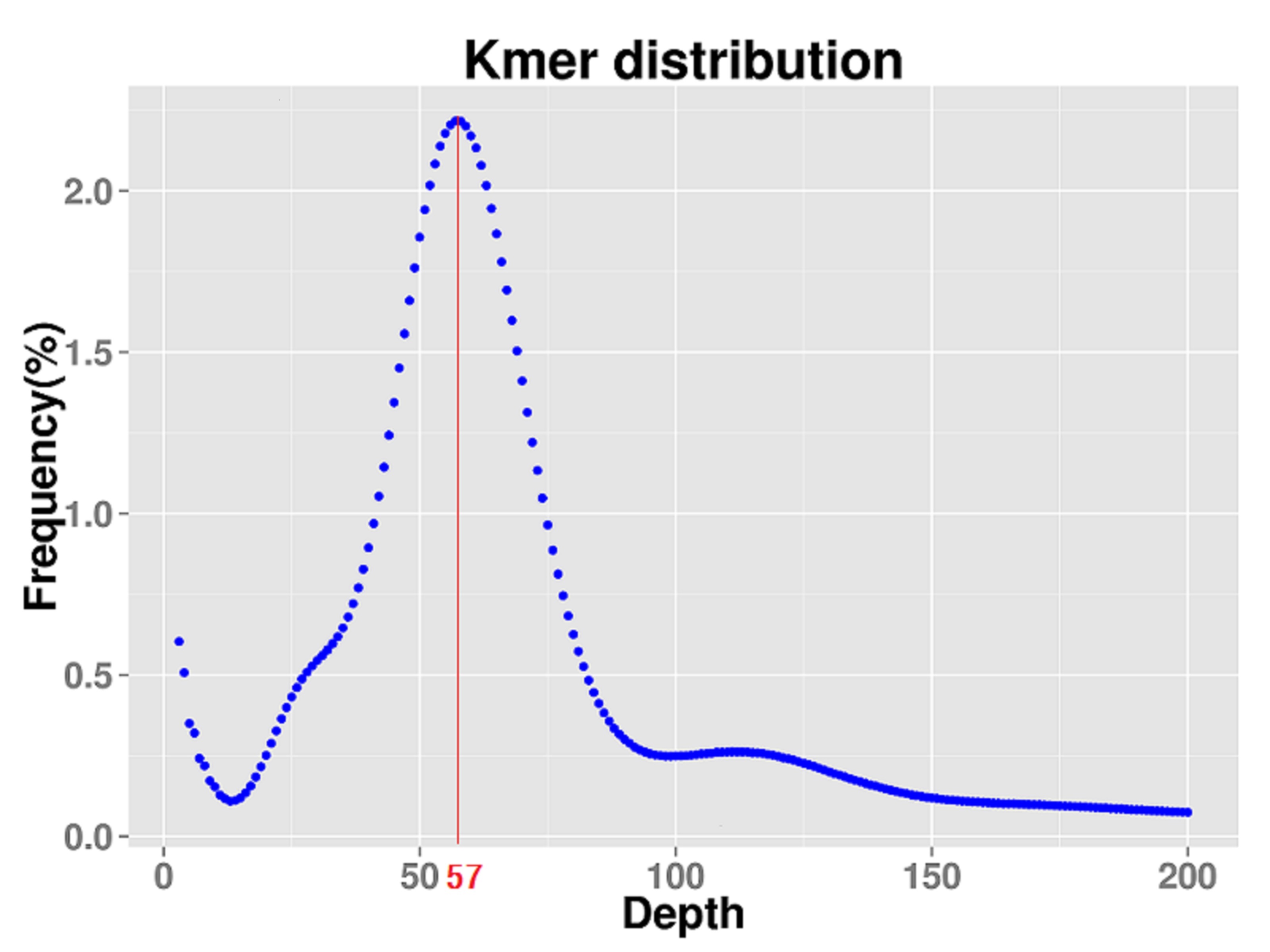

Supplement: Supplementary file 3 — Figure S2 [file 41438_2021_657_MOESM3_ESM.jpg]

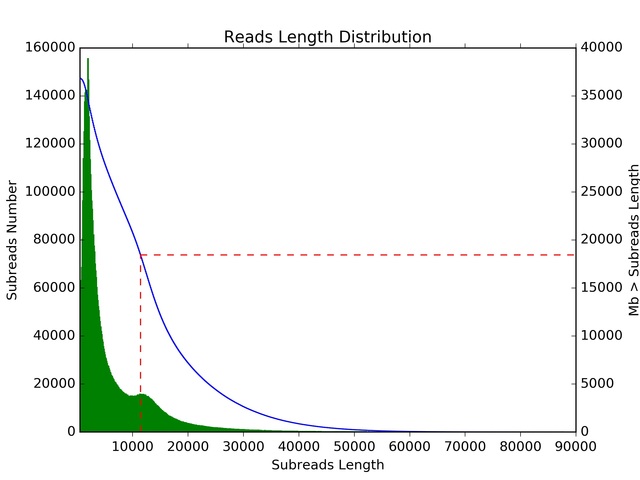

Supplement: Supplementary file 4 — Figure S3 [file 41438_2021_657_MOESM4_ESM.jpg]

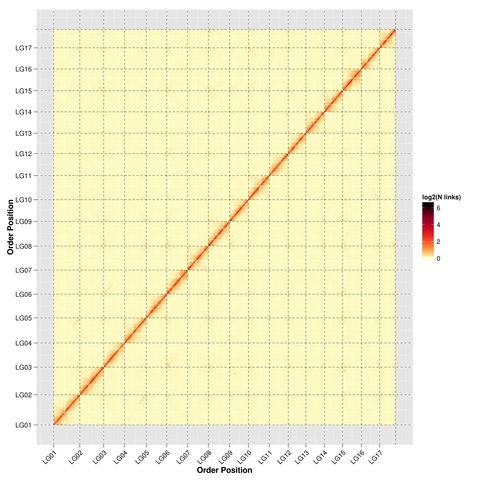

Supplement: Supplementary file 5 — Figure S4 [file 41438_2021_657_MOESM5_ESM.jpg]

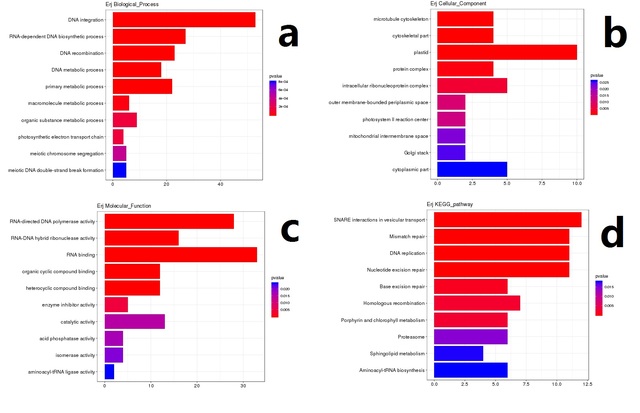

Supplement: Supplementary file 6 — Figure S5 [file 41438_2021_657_MOESM6_ESM.jpg]

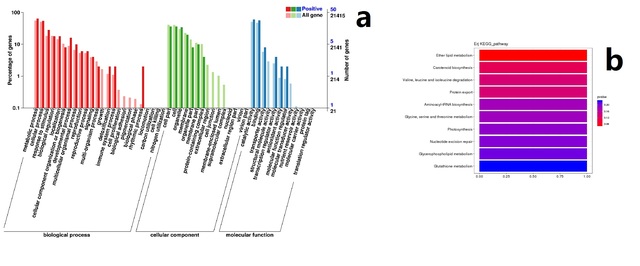

Supplement: Supplementary file 7 — Figure S6 [file 41438_2021_657_MOESM7_ESM.jpg]

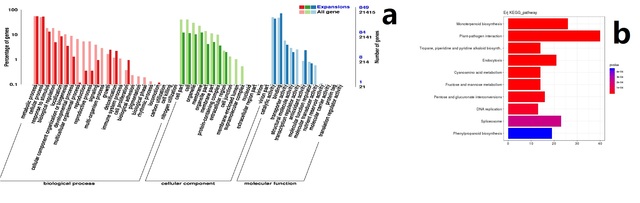

Supplement: Supplementary file 8 — Figure S7 [file 41438_2021_657_MOESM8_ESM.jpg]

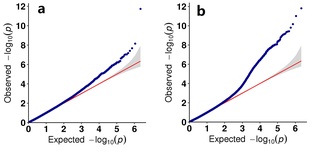

Supplement: Supplementary file 9 — Figure S8 [file 41438_2021_657_MOESM9_ESM.jpg]

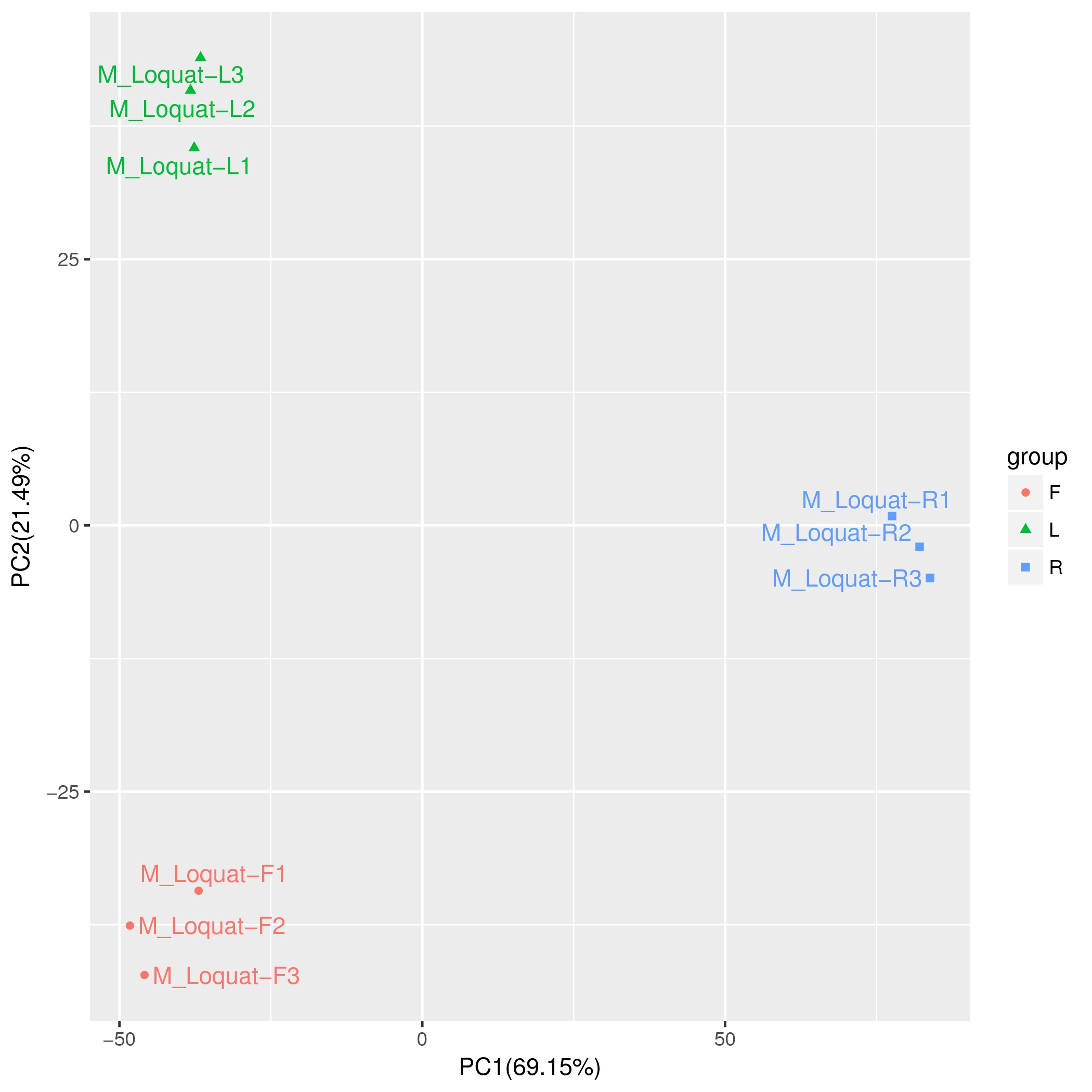

Supplement: Supplementary file 10 — Figure S9 [file 41438_2021_657_MOESM10_ESM.jpg]

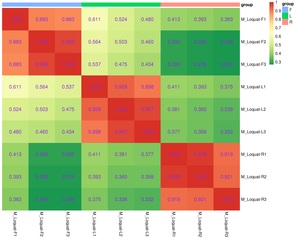

Supplement: Supplementary file 11 — Figure S10 [file 41438_2021_657_MOESM11_ESM.jpg]

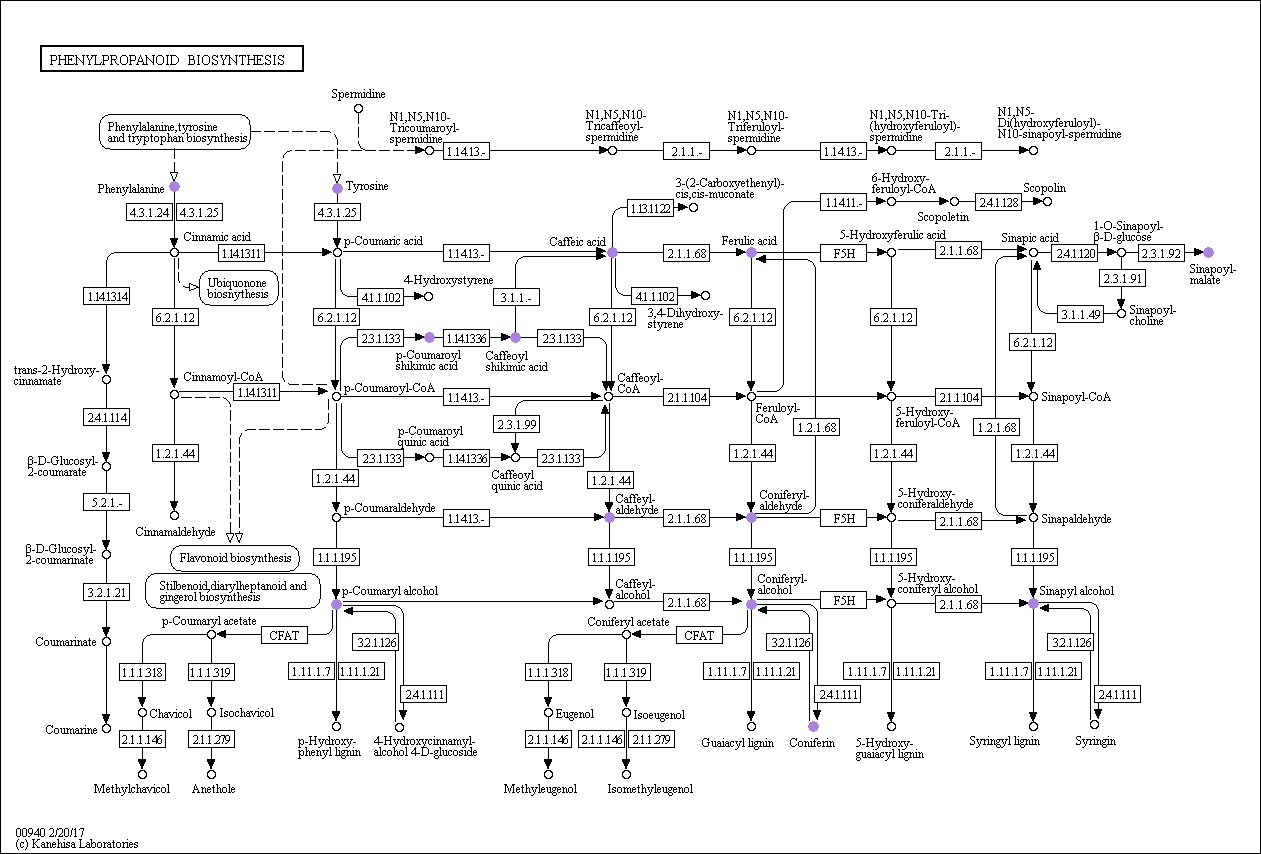

Supplement: Supplementary file 12 — Figure S11 [file 41438_2021_657_MOESM12_ESM.jpg]

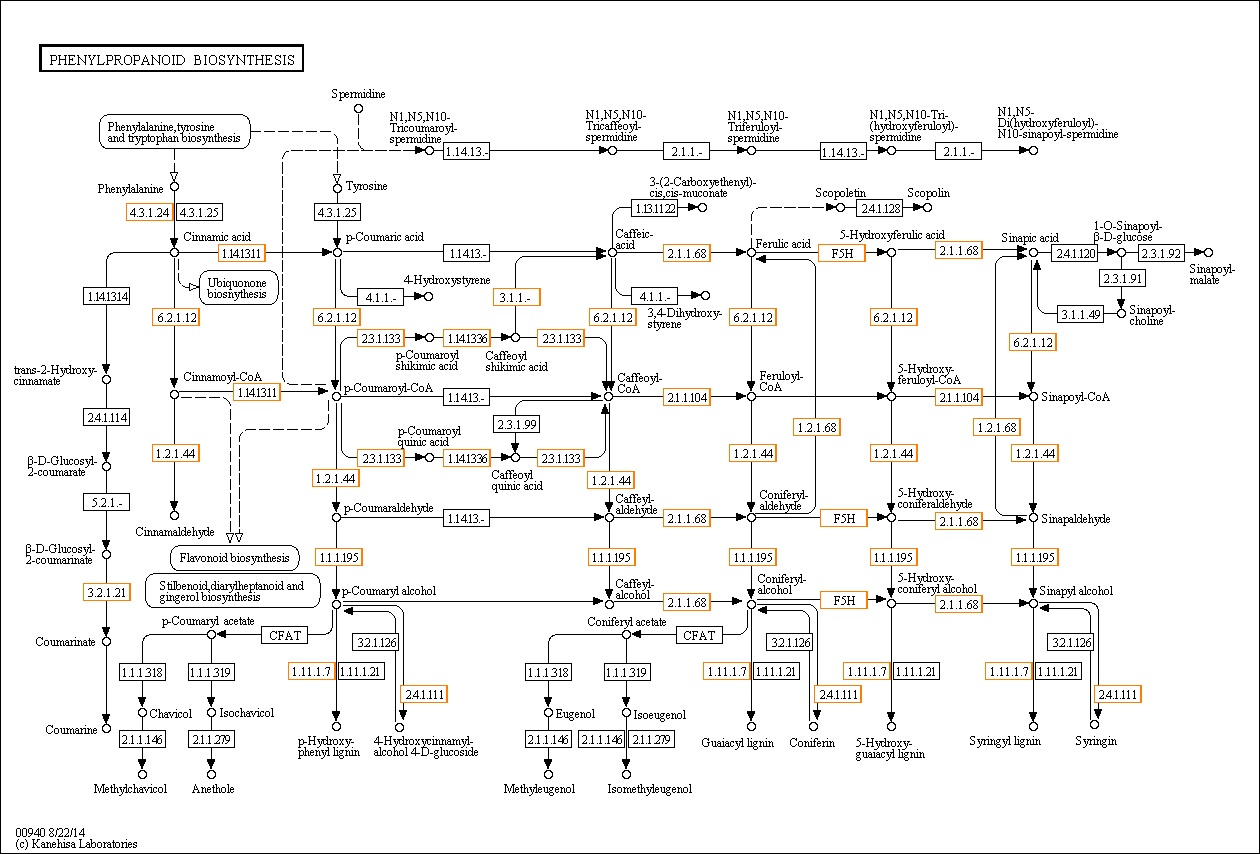

Supplement: Supplementary file 13 — Figure S12 [file 41438_2021_657_MOESM13_ESM.jpg]

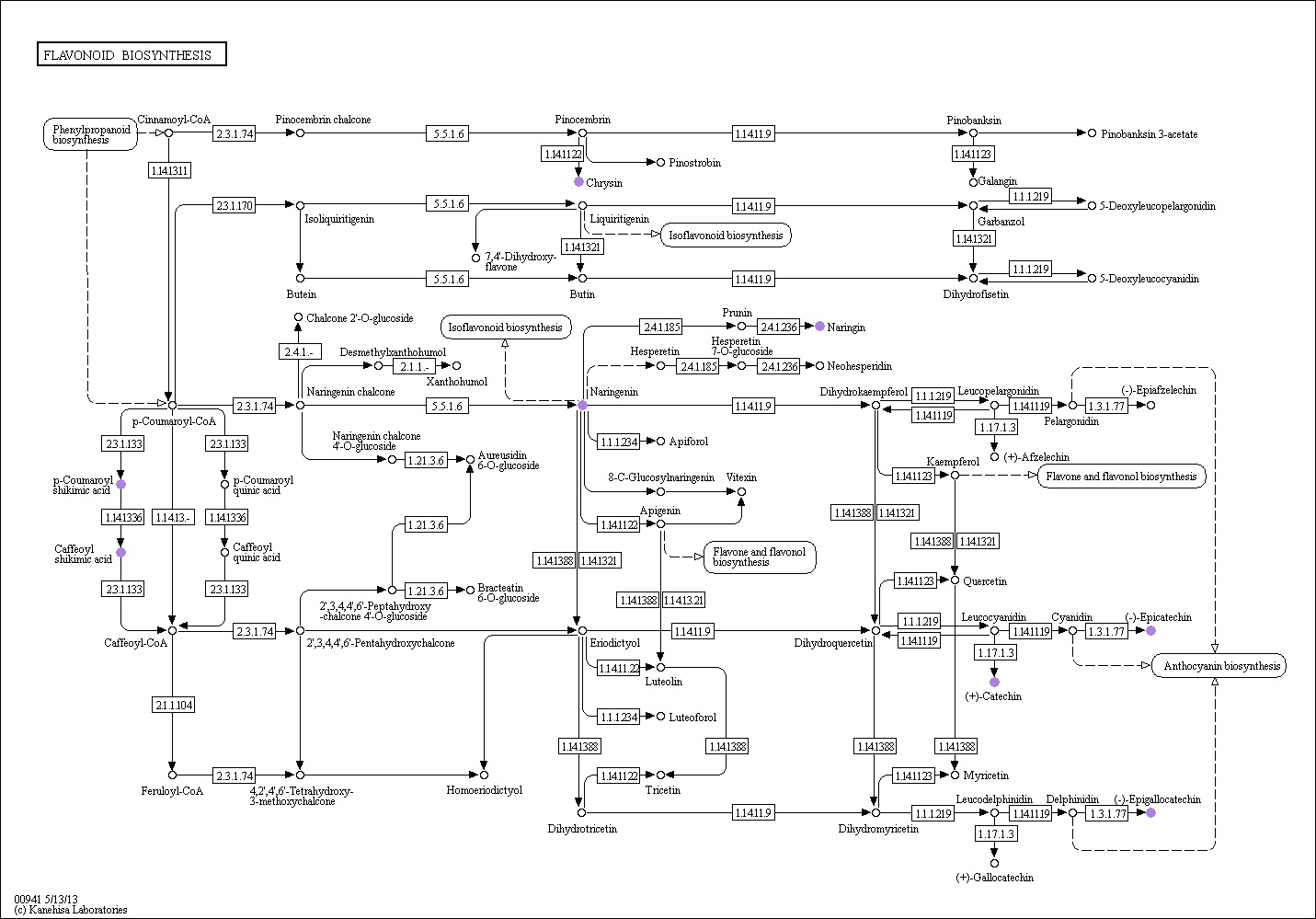

Supplement: Supplementary file 14 — Figure S13 [file 41438_2021_657_MOESM14_ESM.jpg]

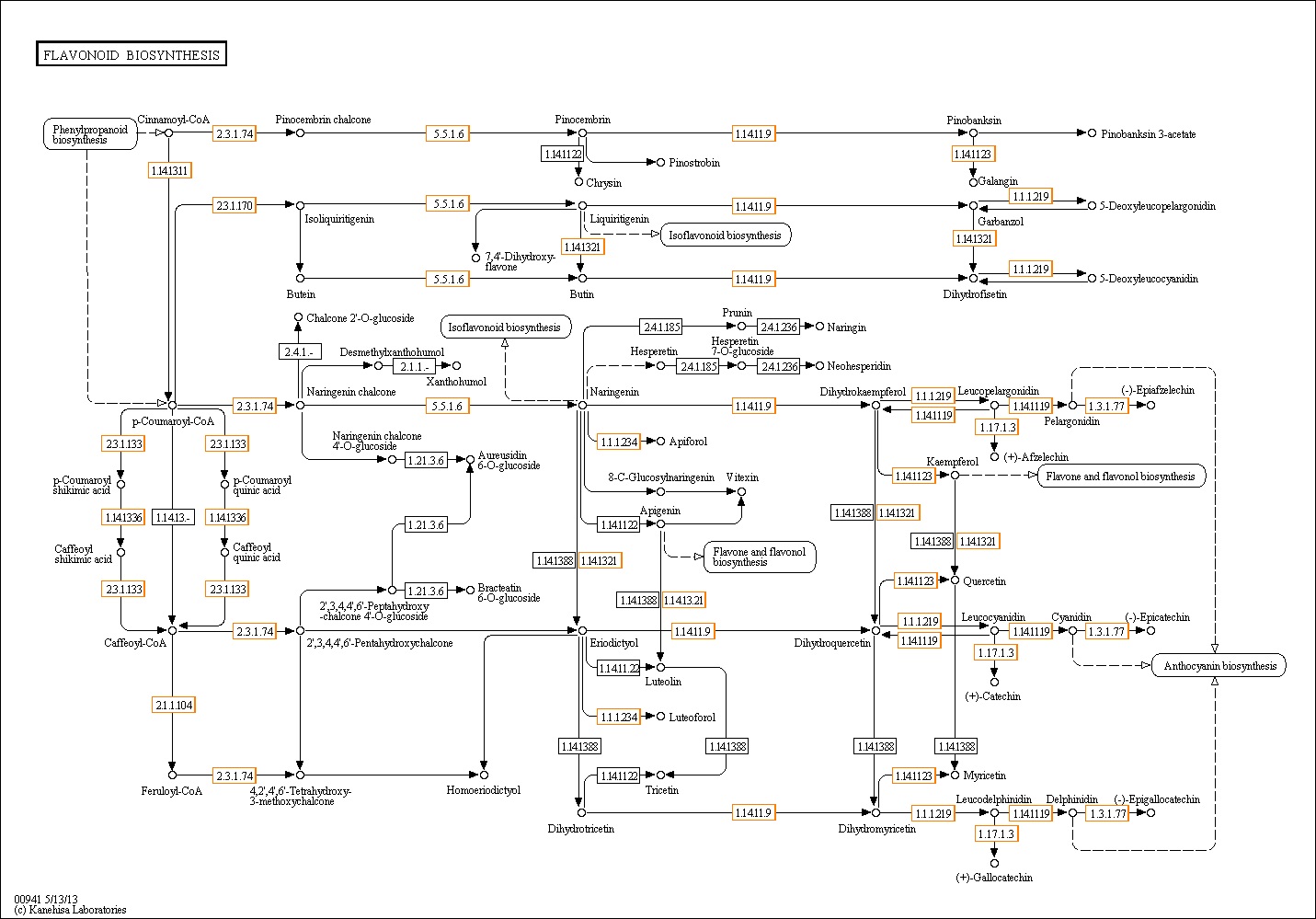

Supplement: Supplementary file 15 — Figure S14 [file 41438_2021_657_MOESM15_ESM.jpg]

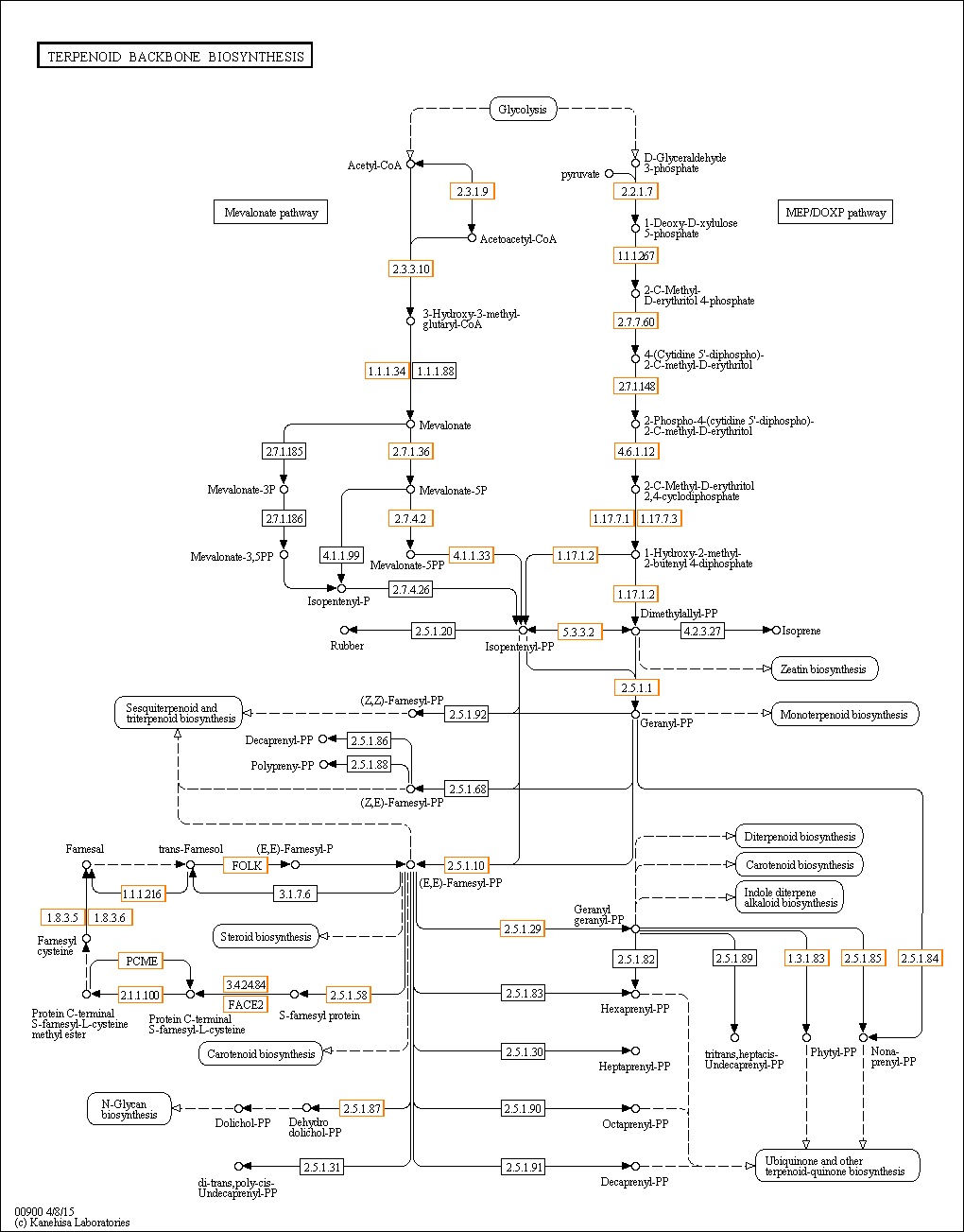

Supplement: Supplementary file 16 — Figure S15 [file 41438_2021_657_MOESM16_ESM.jpg]

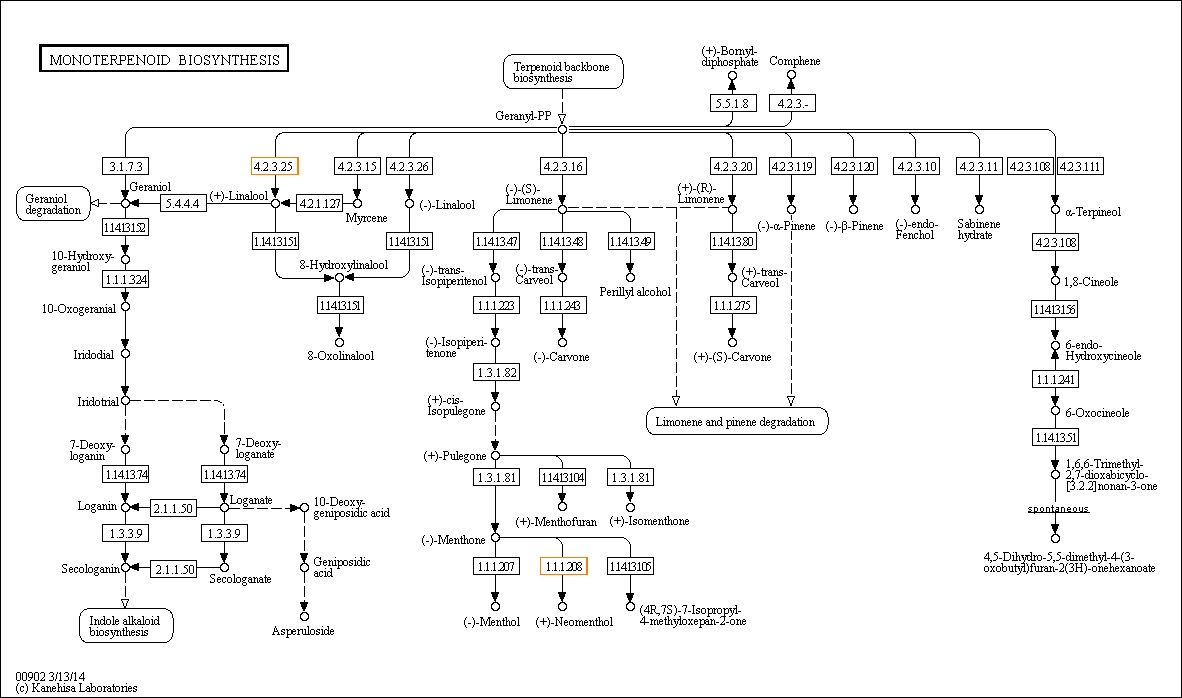

Supplement: Supplementary file 17 — Figure S16 [file 41438_2021_657_MOESM17_ESM.jpg]

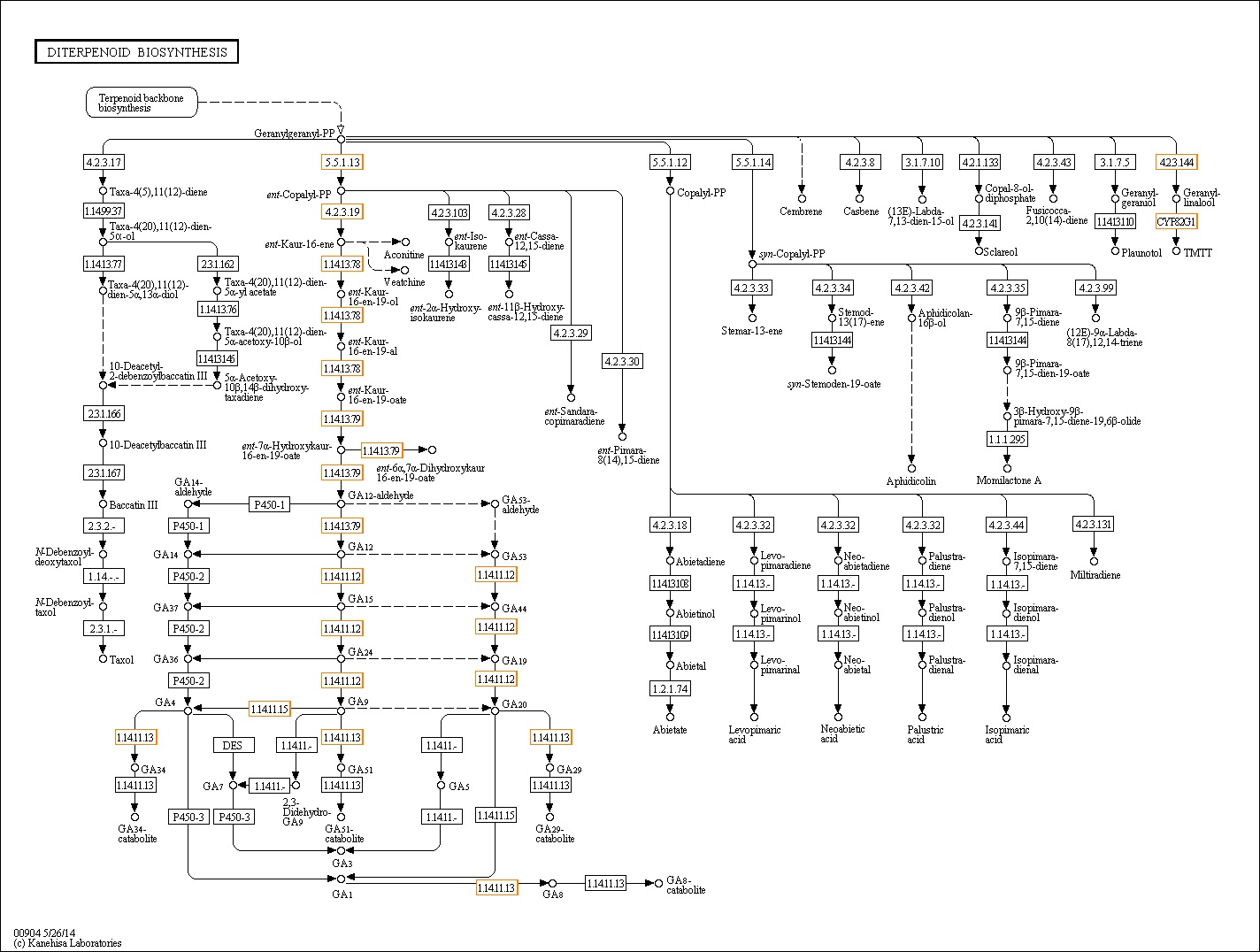

Supplement: Supplementary file 18 — Figure S17 [file 41438_2021_657_MOESM18_ESM.jpg]

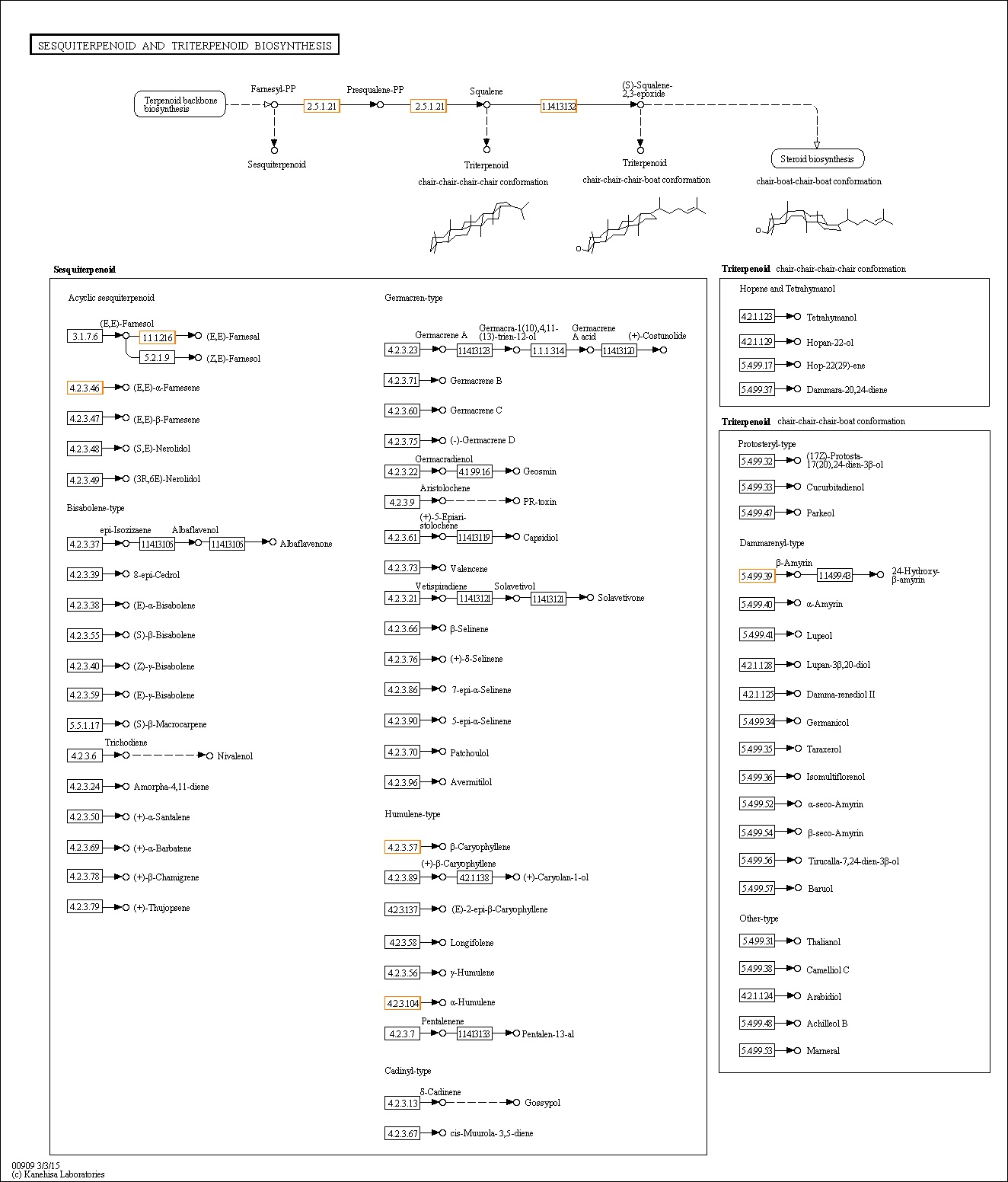

Supplement: Supplementary file 19 — Figure S18 [file 41438_2021_657_MOESM19_ESM.jpg]

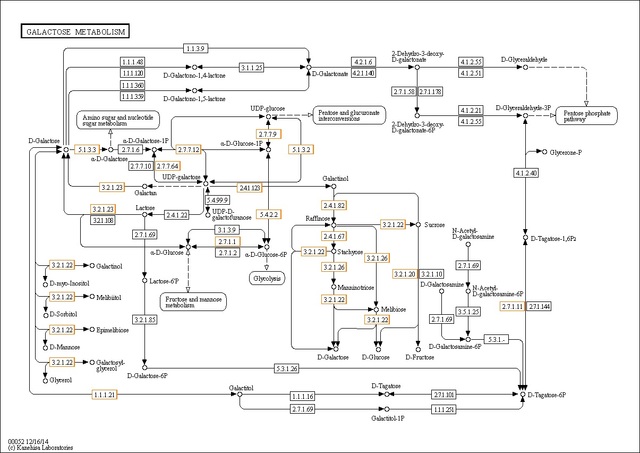

Supplement: Supplementary file 20 — Figure S19 [file 41438_2021_657_MOESM20_ESM.jpg]
